# Easy and direct conversion of tosylates and mesylates into nitroalkanes

**DOI:** 10.3762/bjoc.9.58

**Published:** 2013-03-14

**Authors:** Alessandro Palmieri, Serena Gabrielli, Roberto Ballini

**Affiliations:** 1 “Green Chemistry Group”, School of Science and Technology, Chemistry Division, University of Camerino, Via S. Agostino 1, 62032 Camerino (MC), Italy

**Keywords:** mesylates, nitroalkanes, nucleophilic substitution, tetrabutylammonium nitrite, tosylates

## Abstract

Tosylates and mesylates were directly converted into the corresponding nitroalkanes, by their treatment with tetrabutylammonium nitrite (TBAN) under mild conditions.

## Introduction

**Figure 1:**
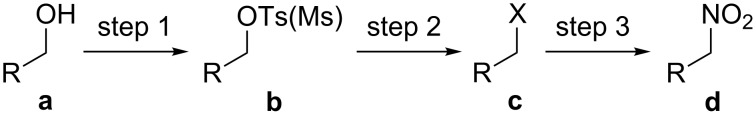
Classical conversion of tosylate and mesylate into the corresponding nitroalkanes via halides.

Nitroalkanes have proven to be one of the most valuable, versatile classes of substances in organic synthesis. In this sense, the chemical literature continuously reports progresses in their utilization for the preparation of a variety of target molecules, such as dyes, plastics, perfumes, pharmaceuticals and many natural products [1–7]. In fact, thanks to the high electron-withdrawing effect of the nitro group, nitroalkanes are prone to afford, under basic conditions, stabilized carbanions, which are commonly used as nucleophiles with a variety of electrophiles [8–11] leading to carbon–carbon bond formation. Furthermore, by making use of the possibility to transform the nitro group into other functionalities, the obtained adducts can be employed as strategic starting materials for the preparation of more complex structures [12–13]. In this context, primary nitroalkanes are the most used nitro derivatives [3–5,14–17], and thus, their availability is crucial. Although these molecules can be obtained from different sources [4], the displacement of alkyl halides with metal nitrite, thanks to the large number of commercially available halides, remains the most used, especially for the primary ones [18–23]. The classical reaction conditions require the usage of MNO_2_ (M = Na, K, Ag) in polar aprotic solvents (DMF, DMSO). Recently, owing to the importance of this transformation, new innovative and more eco-friendly procedures have been developed with success [24–27]. Unfortunately, despite the advantages introduced with these new methodologies, the usefulness of the reaction remains restricted to the use of alkyl halides, and the research of alternative sources is surely welcomed. In this regard, alcohols are a broad class of molecules easily available from both commercial sources and nature and, as a direct source for nitroalkanes, have unsuccessful been explored in the past [28]. In fact, the conversion of alcohols into nitroalkanes requires a three-step sequence, as reported in Figure 1, that necessitates [29–33] (i) conversion of alcohols **a** into their tosylates or mesylates **b**, (ii) transformation of **b** into the corresponding halo derivatives **c** (mainly iodide), and (iii) displacement of the halides by metal nitrite into the nitro compounds **d**.

However, although the first step (**a** into **b**) can be usually performed in quantitative yields, the main drawbacks, in terms of waste production and energy consumption, are in the two-step conversion of **b** into **d**.

Thus, an important advancement could be the development of an easy, direct and mild procedure for transforming tosylates and mesylates into the corresponding nitroalkanes. In fact, with the exception of a couple of examples reporting the conversion of 1-BuOTs [26] and homoallylic tosylates [34] into the corresponding nitro compounds, to the best of our knowledge, no general methods are available in literature.

## Results and Discussion

Following our studies in the development of new processes for the preparation of nitroalkanes [24,27,35], we have now elaborated a general protocol for the direct transformation of aliphatic tosylates and mesylates into the corresponding nitro compounds. In order to optimize the reaction conditions we studied, as a test reaction, the conversion of tosylate **1a** into nitropentadecane **2a** (Table 1). As an initial trial we submitted **1a** to the use of NaNO_2_ as the nitrating agent and DMF as the solvent. Under these conditions, we observed the formation of a mixture of products with traces of compound **2a**. Thus, we switched our attention to a different class of nitrating agent, i.e., the commercially available tetrabutylammonium nitrite (TBAN). The application of 1.5 equiv of this compound in DMF produced **2a** in 27% yield. With this result in our hand we tested both (i) different amounts of TBAN and (ii) different reaction media. As reported in Table 1, we obtained the best result (61% yield) using 1.5 equiv TBAN in toluene as the solvent (Table 1, entry 6).

**Table 1:** Optimization studies.

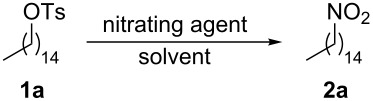				

Entry	Nitrating agent	Solvent^a^	Time (h)	Yield (%)^b^

1	NaNO_2_ (1.5 equiv)	DMF	6	traces
2	Bu_4_NNO_2_ (1.5 equiv)	DMF	6	27
3	Bu_4_NNO_2_ (1.5 equiv)	CPME^c^	6	traces
4	Bu_4_NNO_2_ (1.5 equiv)	Et_2_O	6	traces
5	Bu_4_NNO_2_ (1.5 equiv)	DCM	24	24
6	Bu_4_NNO_2_ (1.5 equiv)	toluene	2.5	61
7	Bu_4_NNO_2_ (1.0 equiv)	toluene	5	45
8	Bu_4_NNO_2_ (2.0 equiv)	toluene	2.5	59

^a^Used 4 mL/mmol. ^b^Yield of pure isolated product. ^c^Cyclopentyl methyl ether.

In order to verify the generality of our method we examined a variety of substrates and, as showed in Table 2, the procedure works well with a variety of primary substrates, including the functionalized ones giving access to a huge typology of primary nitroalkanes in 48–66% overall yields. Moreover the mildness of our reaction conditions permits the survival of important functionalities, such as cyano, ester, chlorine and (*Z*)-C–C double bond, giving easy access to polyfunctionalized nitroalkanes.

**Table 2:** Synthesis of primary nitroalkanes **2a–j**.

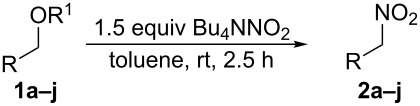				

Entry	R	R^1^	Product	Yield (%)^a^

1	CH_3_(CH_2_)_13_	Ts	**2a**	61
2	CH_3_(CH_2_)_13_	Ms	**2a**	55
3	CH_3_(CH_2_)_8_	Ts	**2b**	65
4	CH_3_(CH_2_)_18_	Ts	**2c**	66
5	CH_3_(CH_2_)_5_	Ts	**2d**	53
6	CH_3_(CH_2_)_5_	Ms	**2d**	50
7	PhCH_2_CH_2_	Ts	**2e**	58
8	PhCH_2_CH_2_	Ms	**2e**	53
9	CH_2_=CH(CH_2_)_8_	Ts	**2f**	60
10	Cl(CH_2_)_5_	Ts	**2g**	59
11	NC(CH_2_)_4_	Ts	**2h**	55
12	AcO(CH_2_)_3_	Ts	**2i**	48
13	(*Z*)-CH_3_(CH_2_)_3_CH=CHCH_2_	Ts	**2j**	57

^a^Yield of pure isolated product.

Finally, we investigated our reaction conditions on secondary substrates, but, as reported in Table 3, the procedure is not general and, for the right substrates, the corresponding nitroalkanes **2k–n** were isolated in lower yields and after longer reaction times with respect to the primary ones.

**Table 3:** Synthesis of secondary nitroalkanes **2k–n**.

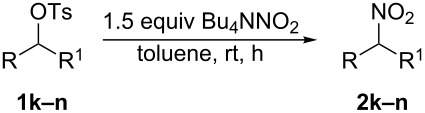				

Entry	R	R^1^	Product	Yield (%)^a^ (Time, h)

1	CH_3_CH_2_	CH_3_(CH_2_)_5_	**2k**	41 (28)
2	CH_3_	PhCH_2_CH_2_	**2l**	33 (39)
3	CH_3_	CH_3_(CH_2_)_5_	**2m**	<10 (37)
4	CH_3_(CH_2_)_2_	CH_3_(CH_2_)_2_	**2n**	<10 (40)

^a^Yield of pure isolated product.

## Conclusion

In conclusion, our method can be considered an important contribution for the preparation of nitroalkanes, making alcohols a strategic source for their availability. Moreover, the mildness of our reaction conditions permits the survival of important functionalities. Furthermore, our protocol presents an interesting peculiarity from the sustainability point of view, since (i) it overcomes the need to convert the alcohol derivatives (tosylates and mesylates) into halides, (ii) low-impacting solvents [36–37] such as toluene and methyl *tert*-butyl ether (work-up) were used, and (iii) any aqueous work-up was avoided, with evident restriction of waste production and energy consumption.

## Experimental

**General Information:** Compounds **2a** [38], **2b** [24], **2c** [15], **2d** [25], **2e** [39], **2f** [24], **2g** [40], **2h** [40], **2i** [24] and **2j** [41] are known, and their spectroscopic data are in agreement with those reported in the literature. Compounds **1** were synthesized by Ghosh and Nicponski’s methodology [42]. ^1^H NMR was recorded at 400 MHz on a Varian Mercury Plus 400. ^13^C NMR was recorded at 100 MHz. Microanalyses were performed with a CHNS-O analyser Model EA 1108 from Fisons Instruments. Mass spectra were performed with a GC–MS system Agilent Technologies 6850 II/5973 Inert by means of the EI technique (70 eV). IR spectra were recorded with a Perkin-Elmer Paragon 500 FTIR.

**General procedure for the conversion of tosylates and mesylates 1 into nitroalkanes 2.** Tetrabutylammonium nitrite (TBAN, 433 mg, 1.5 mmol) was added, at room temperature, to a solution of the appropriate tosylate or mesylate **1** (1 mmol) in toluene (4 mL), and the resulting mixture was stirred at room temperature for 2.5 h (for the reaction time of secondary tosylates, see Table 3). Once the reaction was completed, MTBE (methyl *tert*-butyl ether, 5 mL) was added to the mixture with the formation of two phases. The solvent phase (upper phase) was carefully separated, while the other phase was treated with fresh MTBE (5 mL) and extracted under stirring for 10 min, then, the solvent phase was once again separated (the extraction process was repeated four times). Finally, the combined solution was concentrated under vacuum, to afford the crude product **2**, which was purified by flash column chromatography (heptane/ethyl acetate).

## Supporting Information

All synthesized products were characterized by IR, NMR, GC–MS and elementary analysis. The data is reported in Supporting Information File 1, while copies of ^1^H and ^13^C NMR spectra are attached as Supporting Information File 2.

File 1 Spectroscopic data of synthesized compounds.

File 2 Copy of ^1^H and ^13^C NMR spectra of synthesized compounds.
