# Supplementary material for: Easy and direct conversion of tosylates and mesylates into nitroalkanes
Source: Beilstein J Org Chem. 2013 Mar 14;9:533–6. doi: 10.3762/bjoc.9.58 (PMC3628907; doi:10.3762/bjoc.9.58)
Supplement: File 1 — Spectroscopic data of synthesized compounds. [file Beilstein_J_Org_Chem-09-533-s001.pdf]

Supporting Information

for

Easy and direct conversion of tosylates and mesylates into nitroalkanes

Alessandro Palmieri, Serena Gabrielli and Roberto Ballini*

Address: “Green Chemistry Group”, School of Science and Technology, Chemistry Division, University of Camerino, Via S. Agostino 1, 62032 Camerino (MC), Italy

Email: Roberto Ballini - roberto.ballini@unicam.it

* Corresponding author

Spectroscopic data of synthesized compounds

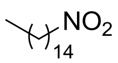

2a. White solid, m.p. = 34–35 °C. IR (nujol) ν: 1381, 1554 cm^{-1}; ^1H NMR (400 MHz, $CDCl_3$) δ: 0.87 (t, 3H, *J* = 6.8 Hz), 1.18–1.43 (m, 24H), 1.94–2.04 (m, 2H), 4.36 (t, 2H, *J* = 7.3 Hz); ^{13}C NMR (100 MHz, $CDCl_3$) δ: 14.3, 22.9, 26.4, 27.6, 29.1, 29.5, 29.6, 29.7, 29.8, 29.9, 32.1, 76.0; MS (EI) *m/z*: 97, 83, 69(100), 57, 55, 43, 41, 29; Anal. calcd for $C_{15}H_{31}NO_2$ (257.41): C, 69.99; H, 12.14; N, 5.44; found: C, 70.03; H, 12.18; N, 5.41.

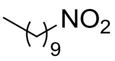

2b. Pale yellow oil. IR (neat) ν: 1381, 1553, 2932 cm^{-1}; ^1H NMR (400 MHz, $CDCl_3$) δ: 0.87 (t, 3H, *J* = 6.8 Hz), 1.18–1.41 (m, 14 H), 1.95–2.04 (m, 2H), 4.37 (t, 2H, *J* = 7.3 Hz); ^{13}C NMR (100 MHz, $CDCl_3$) δ: 14.2, 22.9, 26.3, 27.6, 29.0, 29.2, 29.3, 29.7, 32.0, 75.9; MS (EI) *m/z*: 97, 85, 83, 71, 69, 57, 55(100); Anal. calcd for $C_{10}H_{21}NO_2$ (187.28): C, 64.13; H, 11.30; N, 7.48; found: C, 64,17; H, 11.33; N, 7.47.

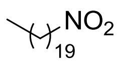

2c. White solid, m.p. = 46–48 °C. IR (nujol) ν: 1379, 1553 cm^{-1}; ^1H NMR (400 MHz, $CDCl_3$) δ: 0.87 (t, 3H, *J* = 6.8 Hz), 1.17–1.41 (m, 34H), 1.95–2.04 (m, 2H), 4.37 (t, 2H, *J* = 7.3 Hz); ^{13}C NMR (100 MHz, $CDCl_3$) δ: 14.4, 22.9, 26.4, 27.6, 29.0, 29.5, 29.6, 29.7, 29.8, 29.9, 30.0, 32.2, 76.0; MS (EI) *m/z*: 97, 83, 69, 57(100), 55, 43, 41, 29; Anal. calcd for $C_{20}H_{41}NO_2$ (327.55): C, 73.34; H, 12.62; N, 4.28; found: C, 73.39; H, 12.66; N, 4.26.

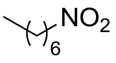

2d. Pale yellow oil. IR (neat) ν: 1379, 1556, 2934 cm^{-1}; ^1H NMR (400 MHz, $CDCl_3$) δ: 0.88 (t, 3H, *J* = 6.8 Hz), 1.18–1.44 (m, 14 H), 1.94–2.05 (m, 2H), 4.37 (t, 2H, *J* = 7.3 Hz); ^{13}C NMR (100 MHz, $CDCl_3$) δ: 14.2, 22.7, 26.43, 27.6, 28.7, 31.7, 76.0; MS (EI) *m/z*: 112, 97, 69, 57, 55(100), 43, 41, 29; Anal. calcd for $C_7H_{15}NO_2$ (145.20): C, 57.90; H, 10.41; N, 9.65; found: C, 57.96; H, 10.37; N, 9.67.

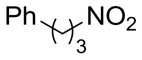

2e. Pale yellow oil. IR (neat) ν: 1382, 1558, 2942, 3025 cm^{-1}. ^1H NMR (400 MHz, $CDCl_3$) δ: 2.30–2.38 (m, 2H), 2.73 (t, 2H, *J* = 7.3 Hz), 4.37 (t, 2H, *J* = 7.3 Hz), 7.18–7.40 (m, 5H); ^{13}C NMR (100 MHz, $CDCl_3$) δ: 29.0, 32.4, 74.8, 126.7, 128.6, 128.9, 139.7; MS (EI) *m/z*: 165(M^+), 117, 104, 91(100), 79, 65; Anal. calcd for $C_9H_{11}NO_2$ (165.19): C, 65.44; H, 6.71; N, 8.48; found: C, 65.42; H, 6.74; N, 8.50.

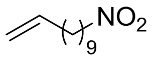

2f. Clear oil. IR (neat) ν: 1381, 1555, 3012 cm^{-1}; ^1H NMR (400 MHz, $CDCl_3$) δ: 1.23–1.44 (m, 12H), 1.94–2.08 (m, 4H), 4.37 (t, 2H, *J* = 7.3 Hz), 4.89–5.02 (m, 2H), 5.73–5.87 (m, 1H); ^{13}C NMR (100 MHz, $CDCl_3$) δ: 26.4, 27.6, 29.0, 29.1, 29.2, 29.4, 29.5, 34.0, 75.9, 114.4, 139.3; MS (EI) *m/z*: 109, 95, 81, 69, 55(100), 41, 29; Anal. calcd for $C_{11}H_{21}NO_2$ (199.29): C, 66.29; H, 10.62; N, 7.03; found: C, 66.34; H, 10.65; N, 7.00.

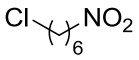

2g. Clear oil. IR (neat) ν: 1383, 1552, 2940 cm^{-1}; ^1H NMR (400 MHz, $CDCl_3$) δ: 1.33–1.53 (m, 4H), 1.70–1.80 (m, 2H), 1.94–2.04 (m, 2H), 3.50 (t, 2H, *J* = 3.4 Hz), 4.36 (t, 2H, *J* = 6.8 Hz); ^{13}C NMR (100 MHz, $CDCl_3$) δ: 25.8, 26.3, 27.4, 32.3, 45.0, 75.7; MS (EI) *m/z*: 130, 83, 81, 55(100), 41, 29; Anal. calcd for $C_6H_{12}ClNO_2$ (165.62): C, 43.51; H, 7.30; N, 8.46; found: C, 43.55; H, 7.32; N, 8.42.

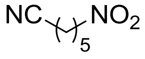

2h. Pale yellow oil. IR (neat) ν: 1377, 1553, 2245, 2933 cm^{-1}; ^1H NMR (400 MHz, $CDCl_3$) δ: 1.48–1.60 (m, 2H), 1.66–1.76 (m, 2H), 1.98–2.09 (m, 2H), 2.37 (t, 2H, *J* = 7.3 Hz), 4.39 (t, 2H, *J* = 6.8 Hz); ^{13}C NMR (100 MHz, $CDCl_3$) δ: 17.1, 24.8, 25.5, 26.6, 75.2, 119.4; MS (EI) *m/z*: 96, 69, 55(100), 41, 29; Anal. calcd for $C_6H_{10}N_2O_2$ (142.16): C, 50.69; H, 7.09; N, 19.71; found: C, 50.73; H, 7.11; N, 19.67.

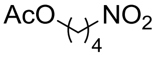

2i. Pale yellow oil. IR (neat) ν: 1378, 1553, 1722, 2928 cm^{-1}; ^1H NMR (400 MHz, $CDCl_3$) δ: 1.67–1.80 (m, 2H), 2.03–2.13 (m, 2H), 2.05 (s, 3 H, CH_3), 4.11 (t, 2H, *J* = 6.4 Hz), 4.42 (t, 2H, *J* = 7.3 Hz,); ^{13}C NMR (100 MHz, $CDCl_3$) δ: 21.0, 24.2, 25.6, 63.3, 75.1, 171.1; MS (EI) *m/z*: 101, 73, 55, 43(100), 29; Anal. calcd for $C_6H_{11}NO_4$ (161.16): C, 44.72; H, 6.88; N, 8.69; found: C, 44.76; H, 6.85; N, 8.66.

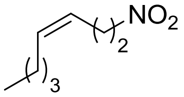

2j. Clear oil. IR (neat) ν: 1378, 1556, 2929, 3016 cm^{-1}; ^1H NMR (400 MHz, $CDCl_3$) δ: 0.89 (t, 3H, *J* = 7.3 Hz), 1.25–1.38 (m, 4H), 1.99–2.10 (m, 2H), 2.74 (q, 2H, *J* = 7.3 Hz), 4.37 (t, 2H, *J* = 7.3 Hz), 5.23–5.33 (m, 1H), 5.52–5.63 (m, 1H); ^{13}C NMR (100 MHz, $CDCl_3$) δ: 14.2, 22.5, 25.7, 27.2, 31.8, 75.3, 122.3, 135.1; MS (EI) *m/z*: 110, 81, 67, 54, 41(100), 29; Anal. calcd for $C_8H_{15}NO_2$ (157.21): C, 61.12; H, 9.62; N, 8.91; found: C, 61.16; H, 9.66; N, 8.88.

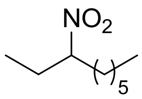

2k. Pale yellow oil. IR (neat) ν: 1379, 1554, 2931 cm^{-1}; ^{1}H NMR (400 MHz, $CDCl_3$) δ: 0.84–0.89 (m, 3H), 0.92–0.97 (m, 3H), 1.17–1.40 (m, 8H), 1.61–1.84 (m, 2H), 1.87–2.03 (m, 2H), 4.34–4.43 (m, 1H); ^{13}C NMR (100 MHz, $CDCl_3$) δ: 10.5, 14.2, 22.7, 26.0, 27.4, 28.8, 31.7, 33.8, 90.7; MS (EI) *m*/*z*: 85, 71, 69, 57, 55, 43, 41(100), 29; Anal. calcd for $C_9H_{19}NO_2$ (173.25): C, 62.39; H, 11.05; N, 8.08; found: C, 62.44; H, 11.09; N, 8.04.

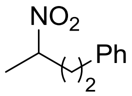

2l. Pale yellow oil. IR (neat) ν: 1379, 1554, 2931 cm^{-1}. ^{1}H NMR (400 MHz, $CDCl_3$) δ: 1.56 (d, 3H, *J* = 6.4 Hz), 1.94–2.06 (m, 1H), 2.31–2.43 (m, 1H), 2.57–2.74 (m, 2H), 4.52–4.62 (m, 1H), 7.16–7.35 (m, 5H); ^{13}C NMR (100 MHz, $CDCl_3$) δ: 19.6, 32.1, 37.0, 82.9, 126.7, 128.7, 128.9, 140.1; MS (EI) *m*/*z*: 107, 91(100), 79, 65; Anal. calcd for $C_{10}H_{13}NO_2$ (179.22): C, 67.02; H, 7.31; N, 7.82; found: C, 66.97; H, 7.27; N, 7.87.

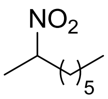

2m. Pale yellow oil. IR (neat) ν: 1378, 1555, 2933 cm^{-1}; ^{1}H NMR (400 MHz, $CDCl_3$) δ: 0.82 (t, 3H, *J* = 7.3 Hz), 1.18–1.33 (m, 8H), 1.55 (d, 3H, *J* = 6.4 Hz), 1.93–2.02 (m, 2H), 4.49–4.57 (m, 1H); ^{13}C NMR (100 MHz, $CDCl_3$) δ: 14.1, 22.2, 22.9, 26.0, 28.9, 30.5, 37.6, 87.1; Anal. calcd for $C_8H_{17}NO_2$ (159.23): C, 60.35; H, 10.76; N, 8.80; found: C, 60.41; H, 10.79; N, 8.75.

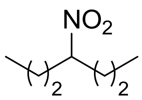

2n. Pale yellow oil. IR (neat) ν: 1377, 1553, 2936 cm^{-1}; ^{1}H NMR (400 MHz, $CDCl_3$) δ: 0.88 (t, 6H, *J* = 7.3 Hz), 1.20–1.31 (m, 4H), 1.88–2.07 (m, 4H), 4.33–4.44 (m, 1H); ^{13}C NMR (100 MHz, $CDCl_3$) δ: 13.8, 20.2, 35.2, 88.1; Anal. calcd for $C_7H_{15}NO_2$ (145.20): C, 57.90; H, 10.41; N, 9.65; found: C, 57.95; H, 10.45; N, 9.61.
