# Supplementary material for: Easy and direct conversion of tosylates and mesylates into nitroalkanes
Source: Beilstein J Org Chem. 2013 Mar 14;9:533–6. doi: 10.3762/bjoc.9.58 (PMC3628907; doi:10.3762/bjoc.9.58)

Supporting Information

for

Easy and direct conversion of tosylates and mesylates into nitroalkanes

Alessandro Palmieri, Serena Gabrielli and Roberto Ballini*

Address: “Green Chemistry Group”, School of Science and Technology, Chemistry Division, University of Camerino, Via S. Agostino 1, 62032 Camerino (MC), Italy

Email: Roberto Ballini - roberto.ballini@unicam.it

* Corresponding author

Copy of ^{1}H and ^{13}C NMR spectra of synthesized compounds

^{1}H NMR - Compound **2a**.

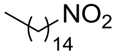

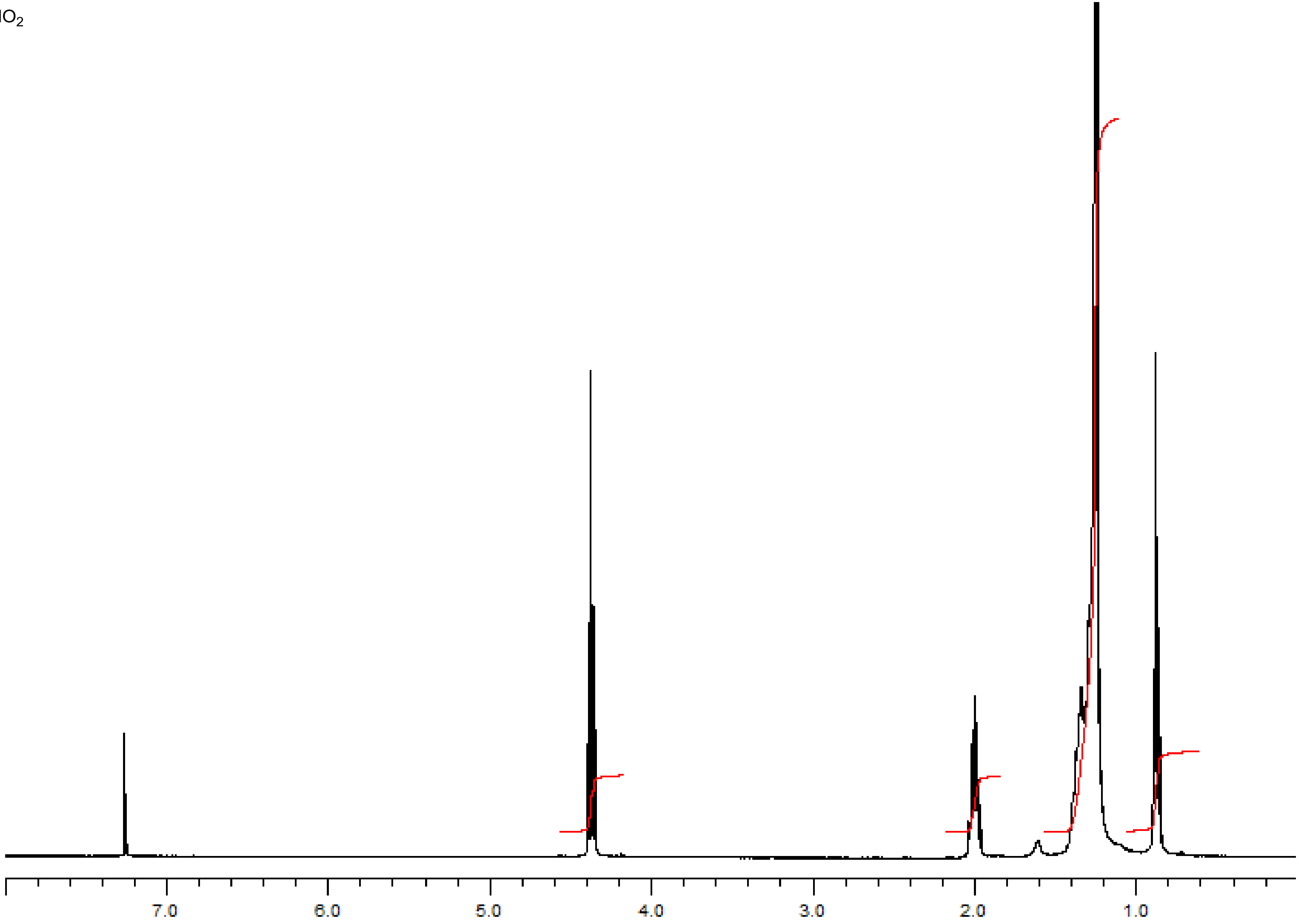

^{13}C NMR - Compound **2a**.

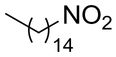

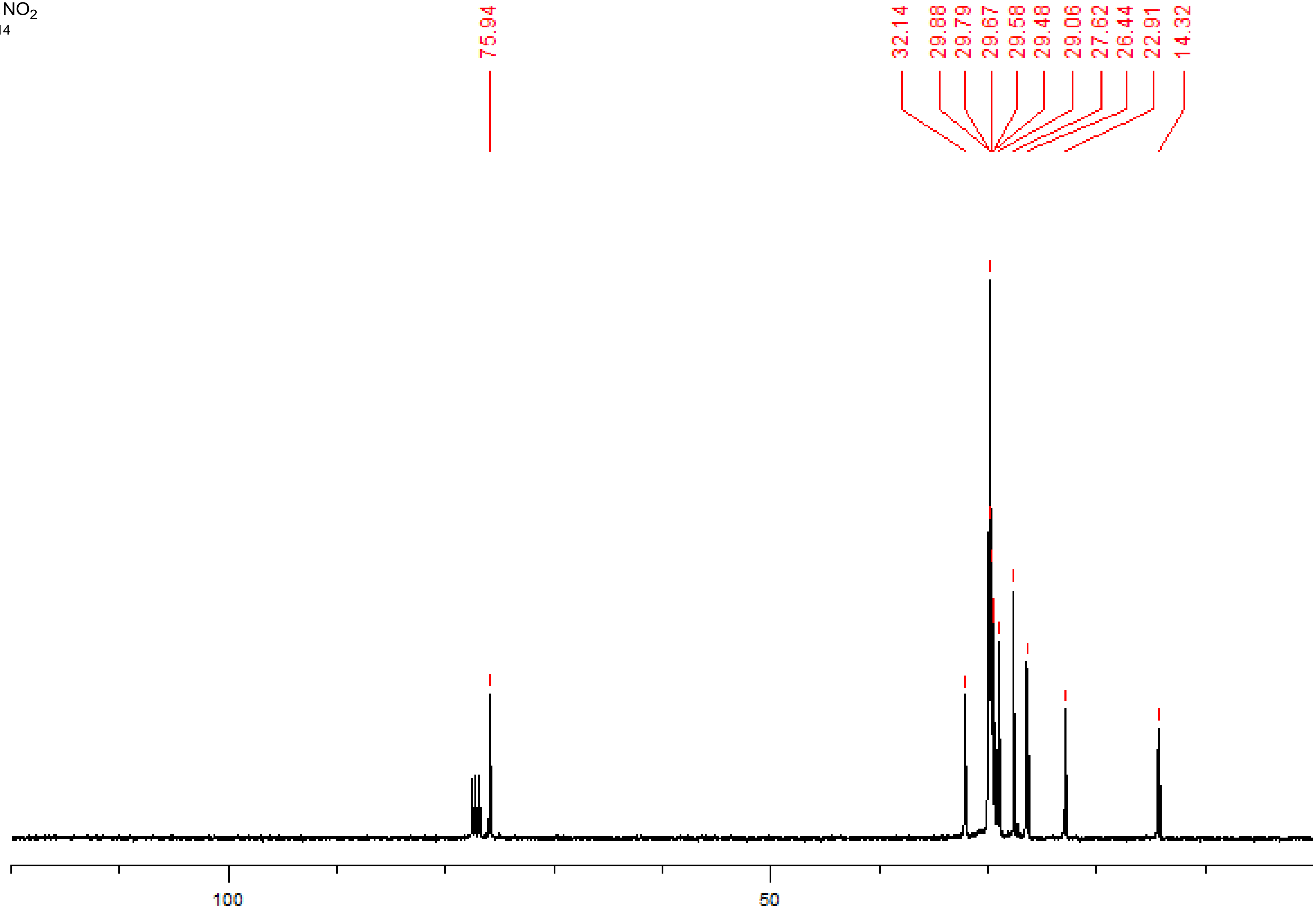

^{1}H NMR - Compound **2b**.

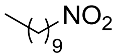

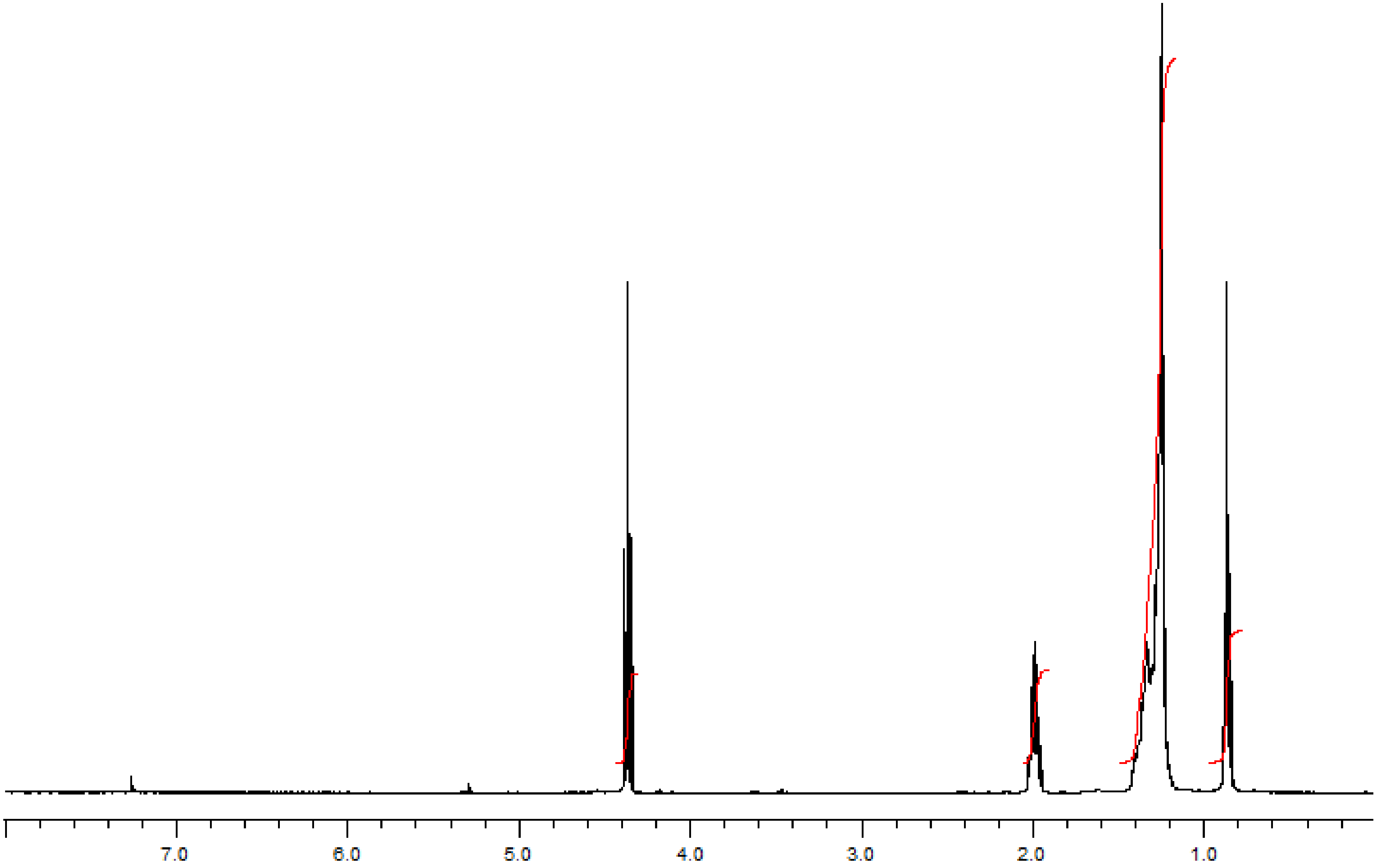

^{13}C NMR - Compound **2b**.

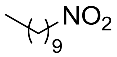

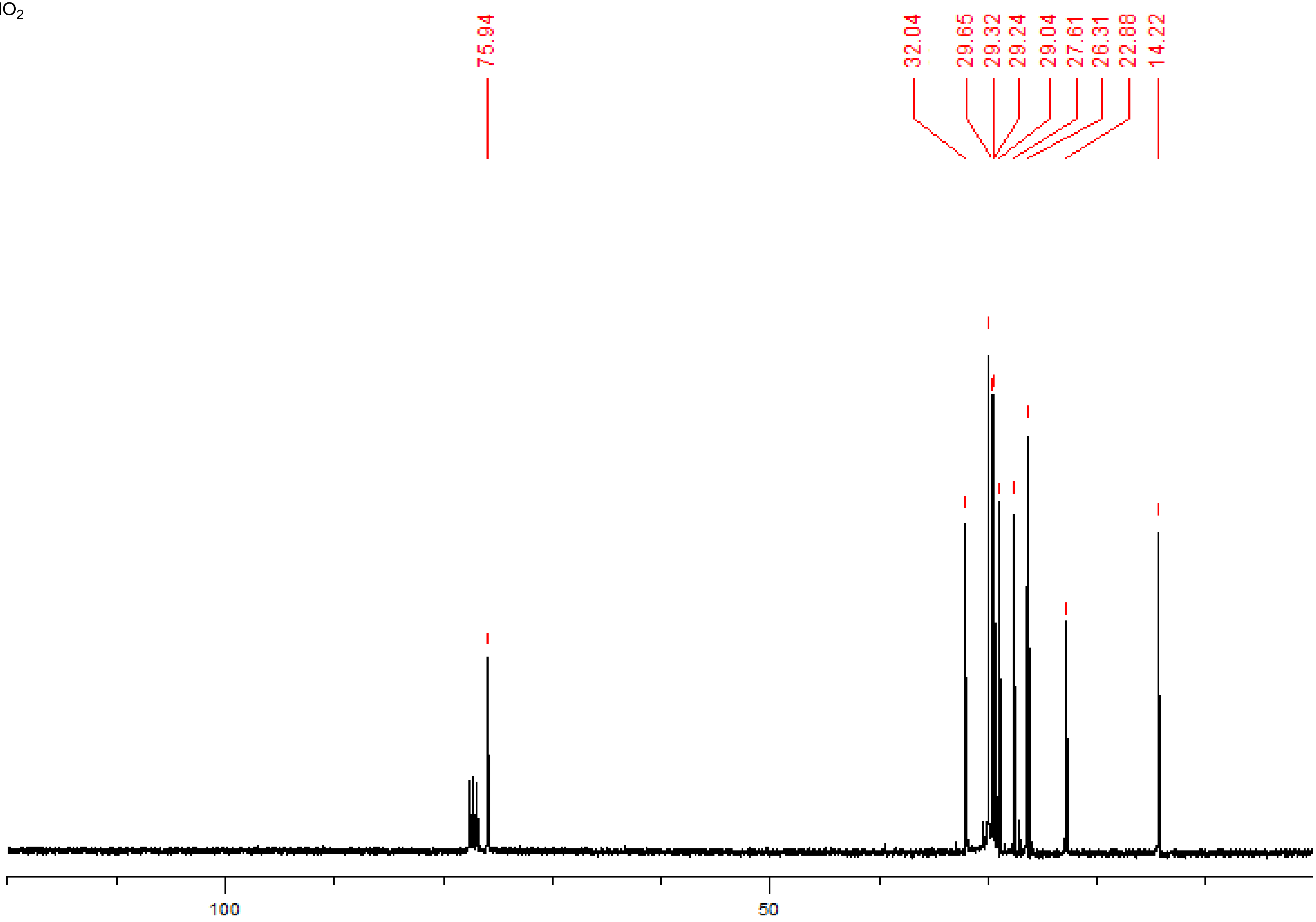

^{1}H NMR - Compound **2c**.

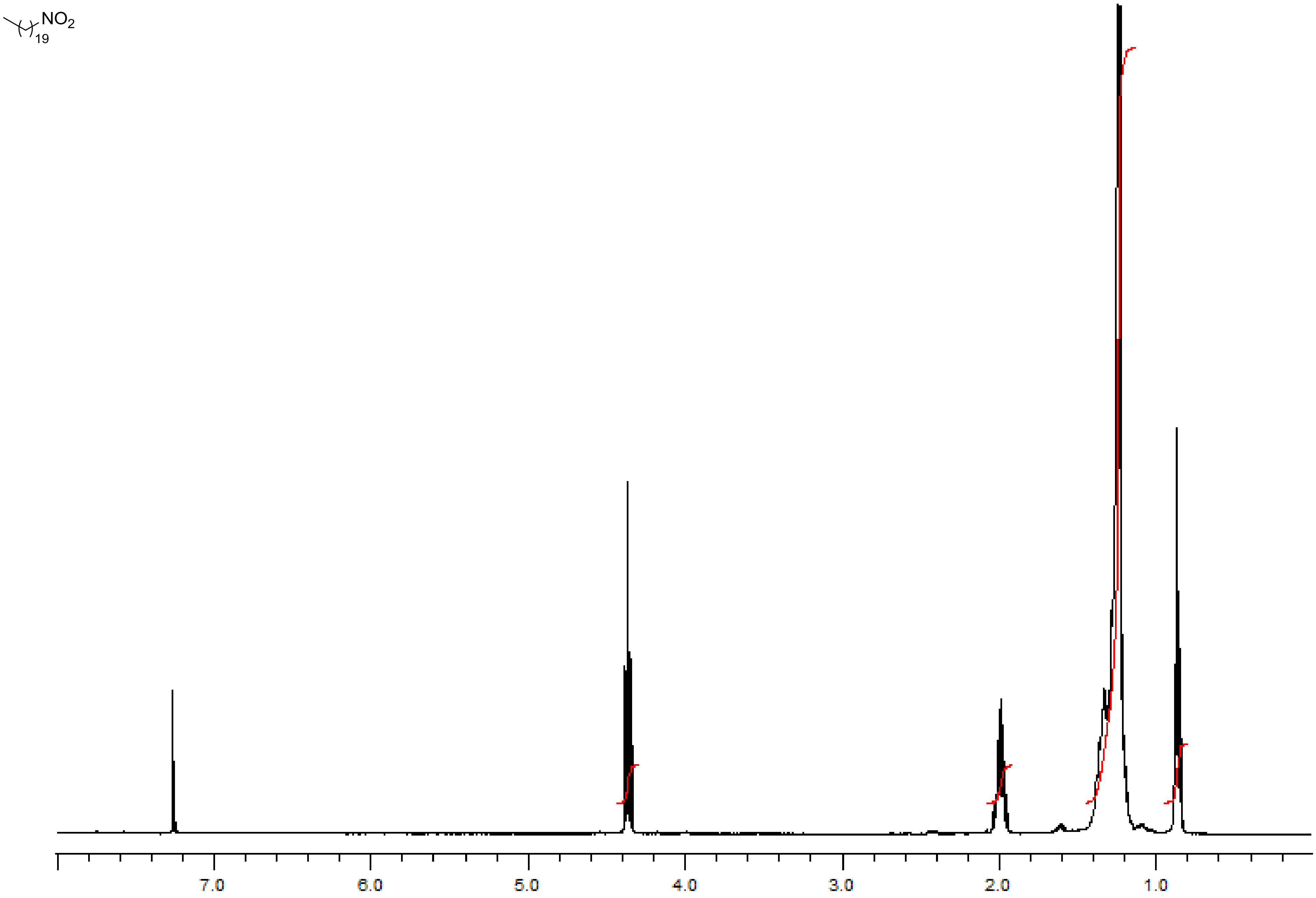

^{13}C NMR - Compound **2c**.

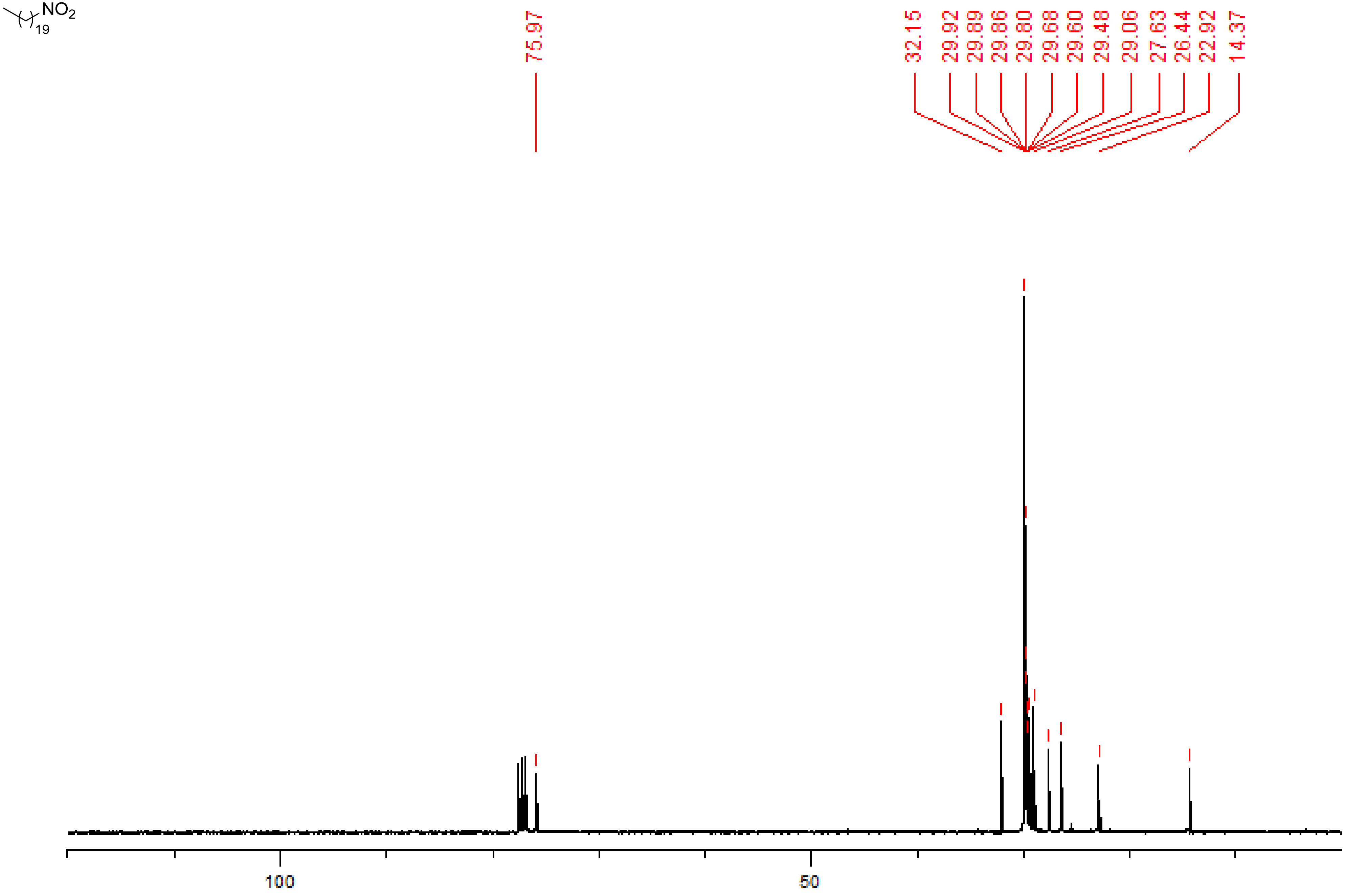

^{1}H NMR - Compound **2d**.

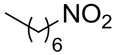

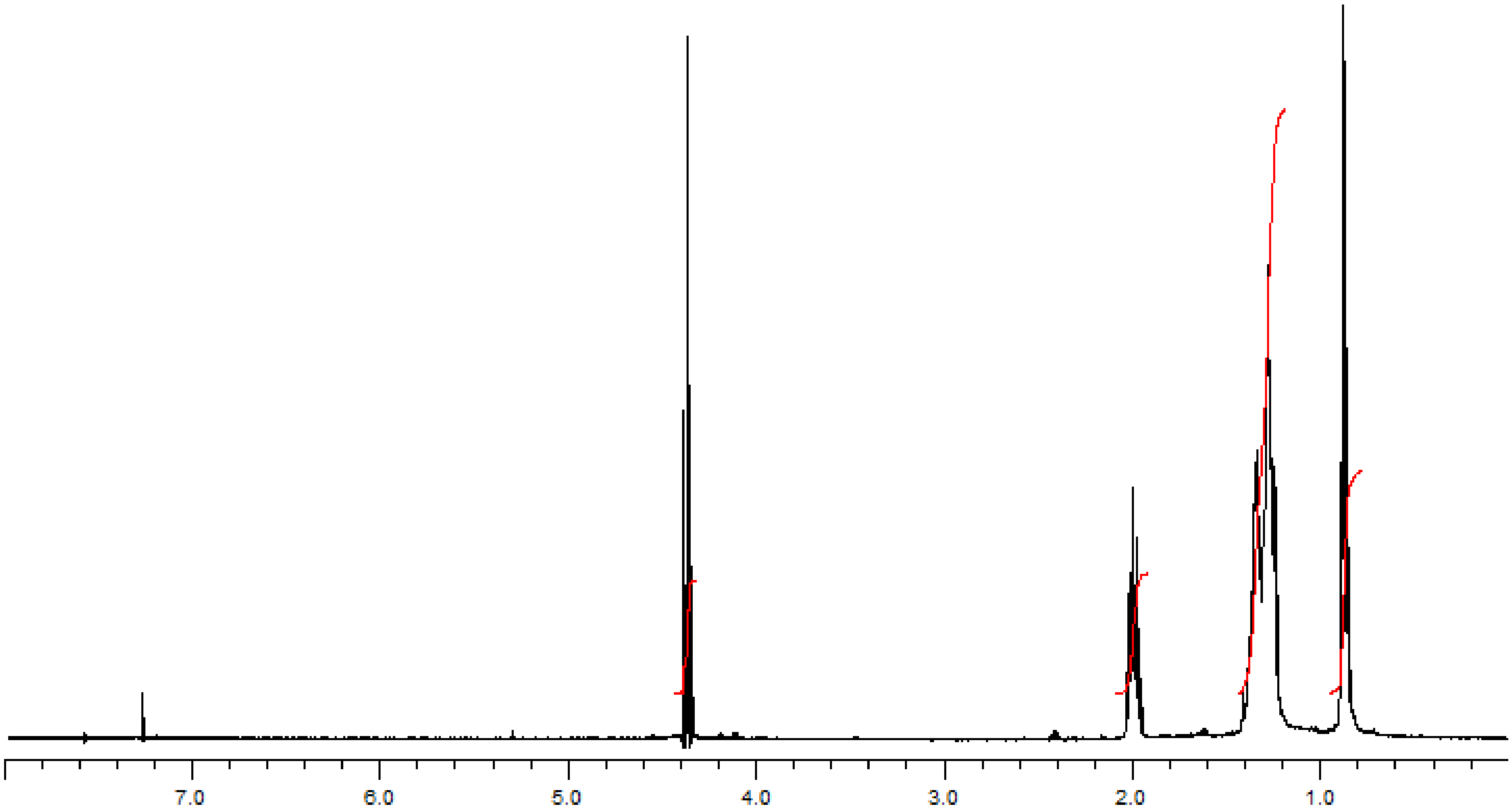

^{13}C NMR - Compound **2d**.

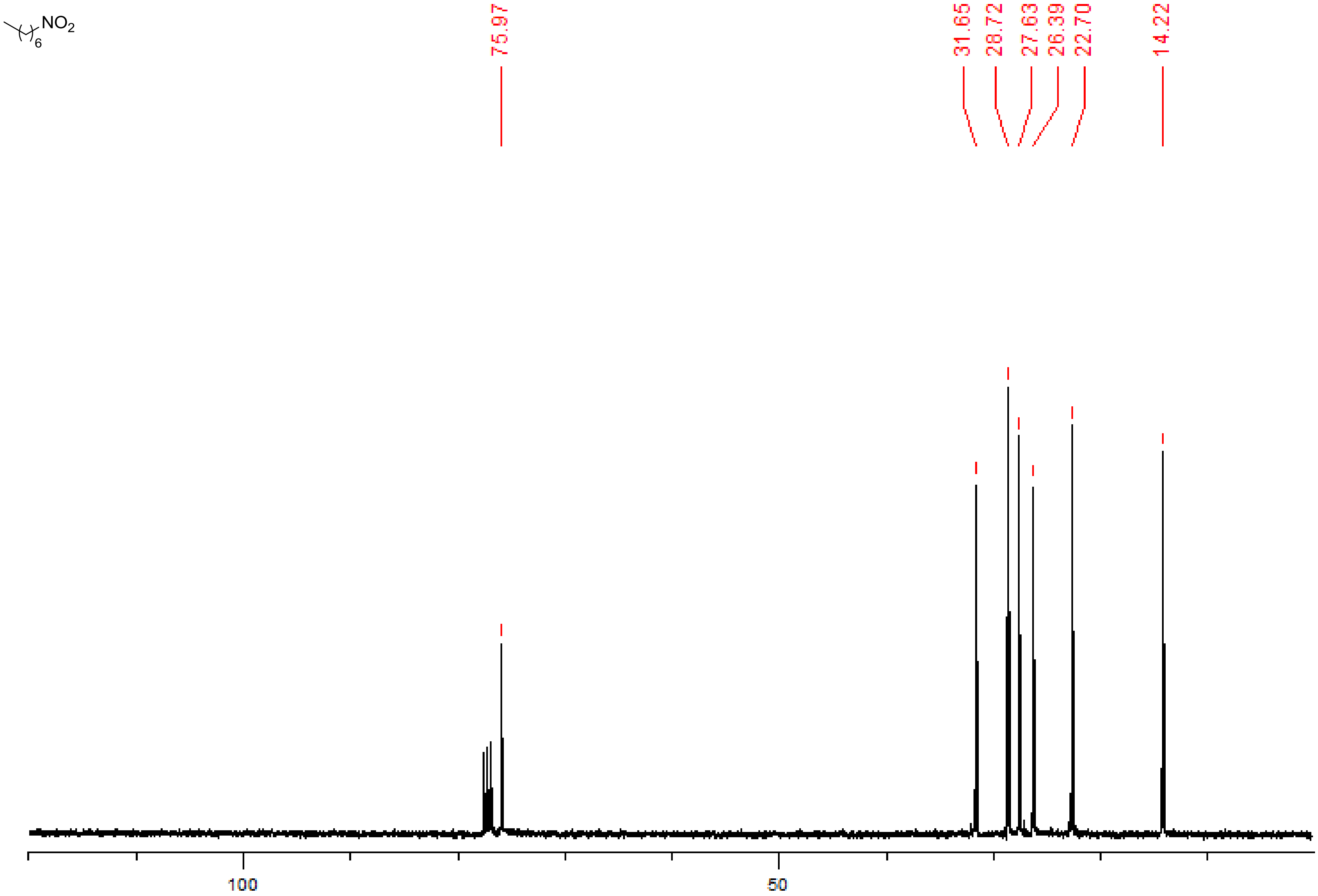

^{1}H NMR - Compound **2e**.

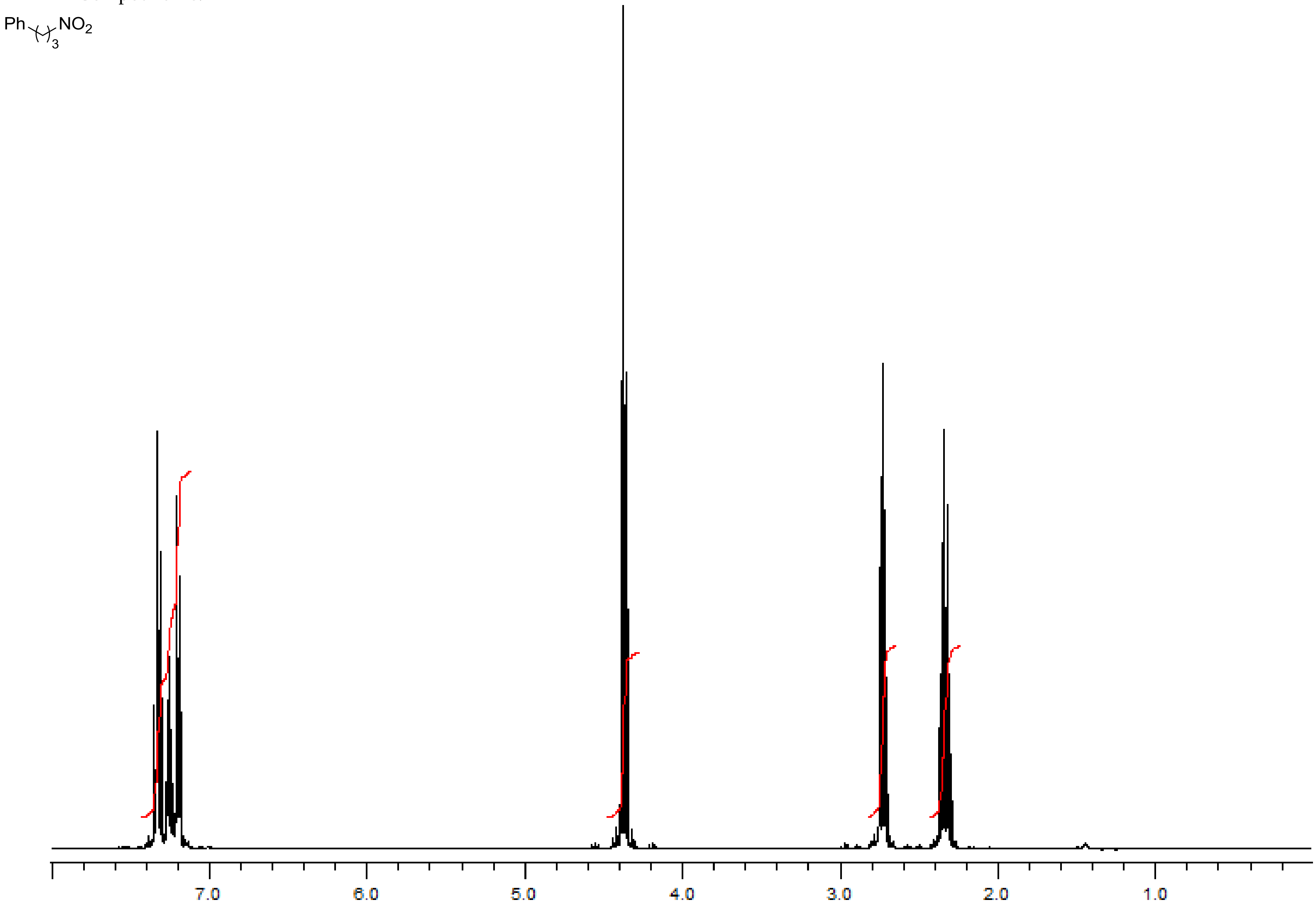

^{13}C NMR - Compound **2e**.

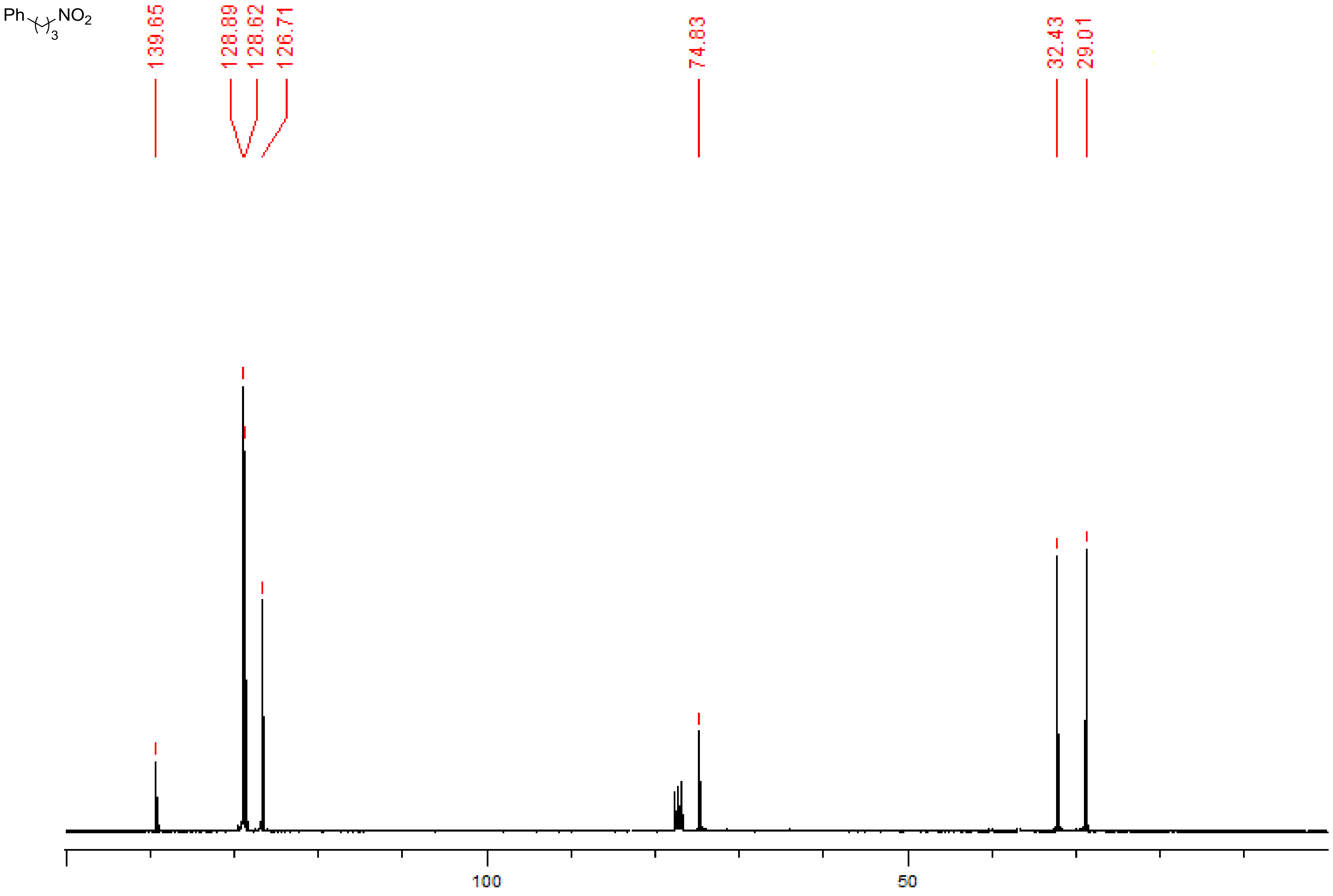

^{1}H NMR - Compound **2f**.

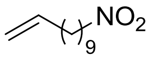

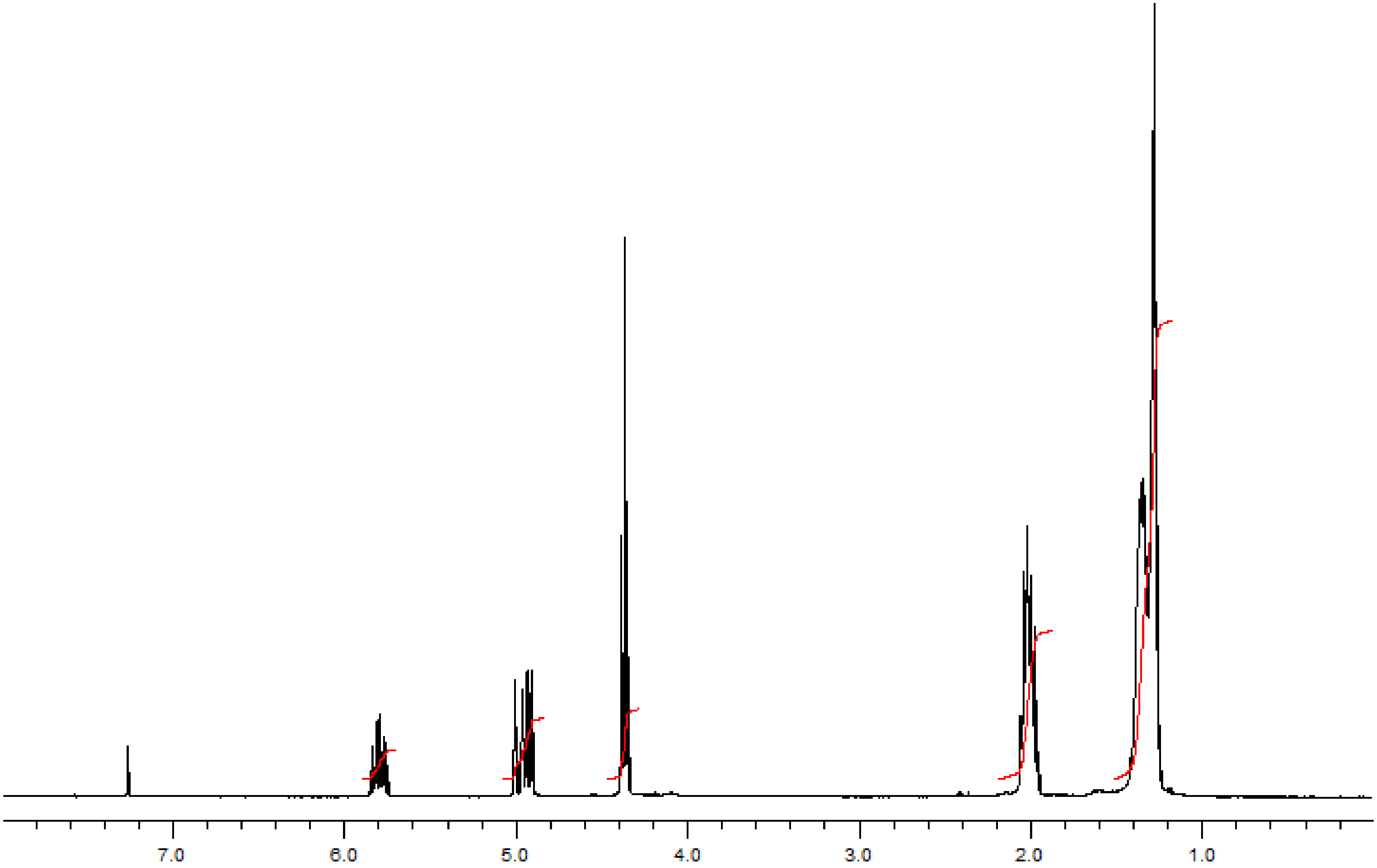

^{13}C NMR - Compound **2f**.

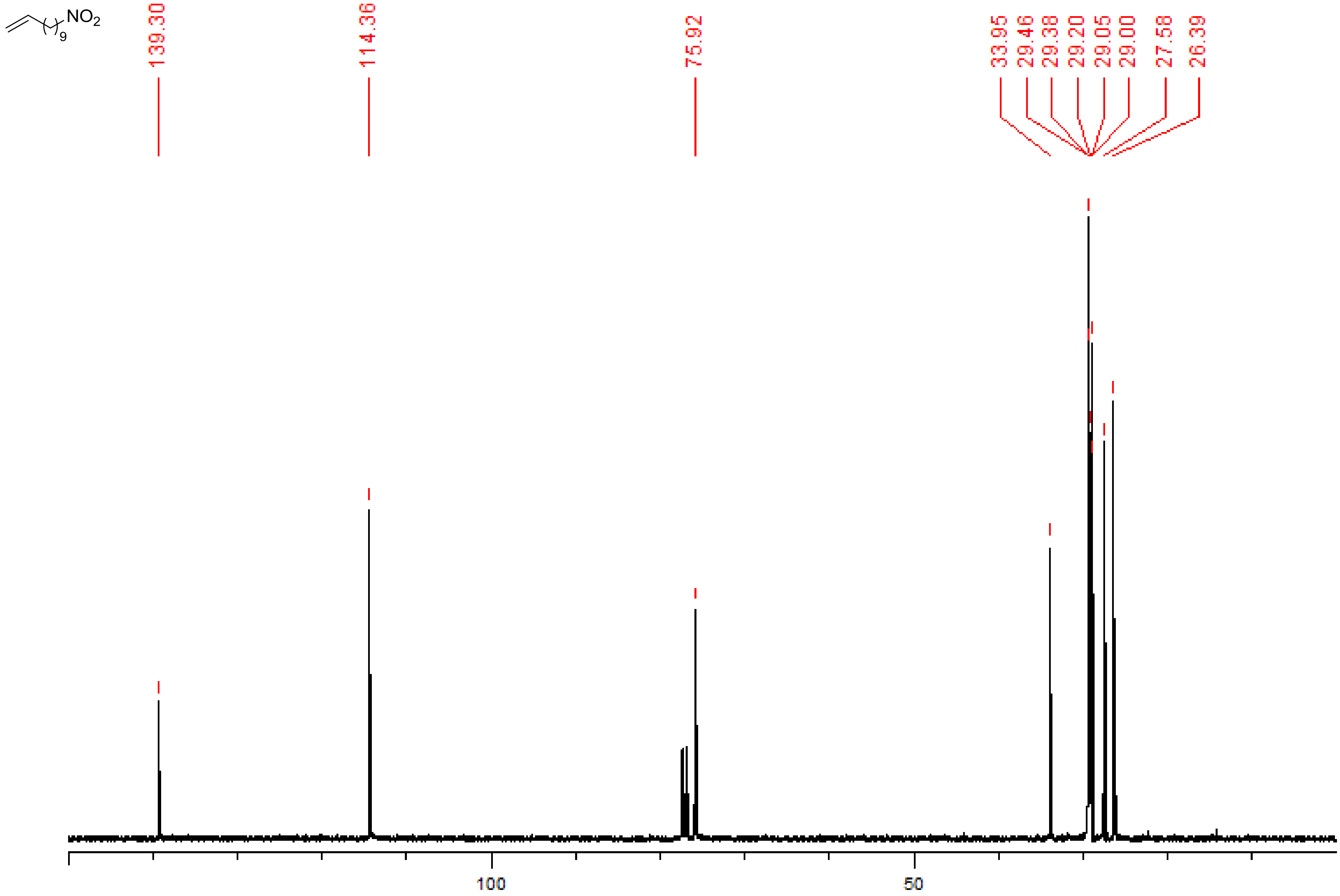

^{1}H NMR - Compound **2g**.

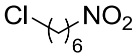

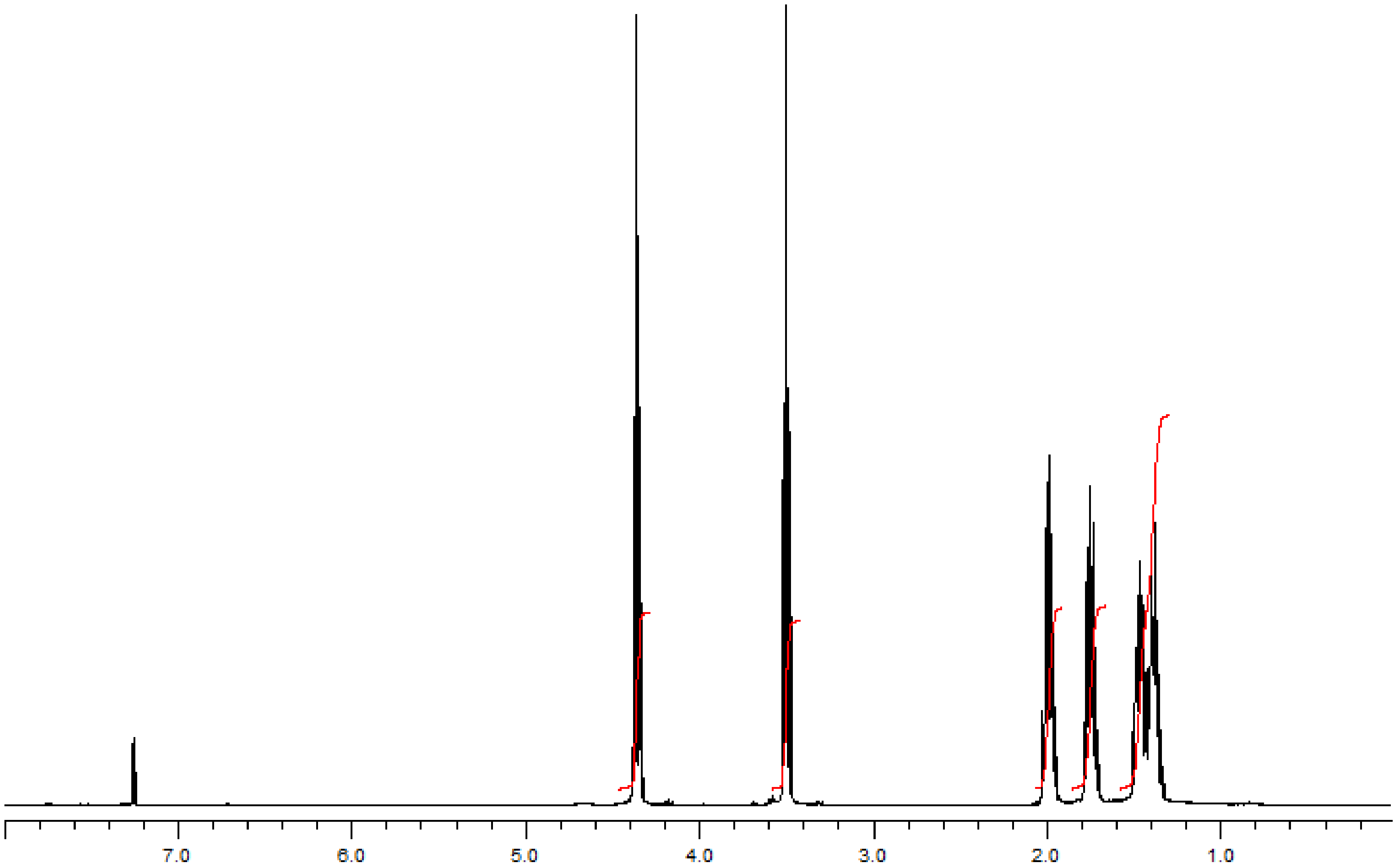

^{13}C NMR - Compound **2g**.

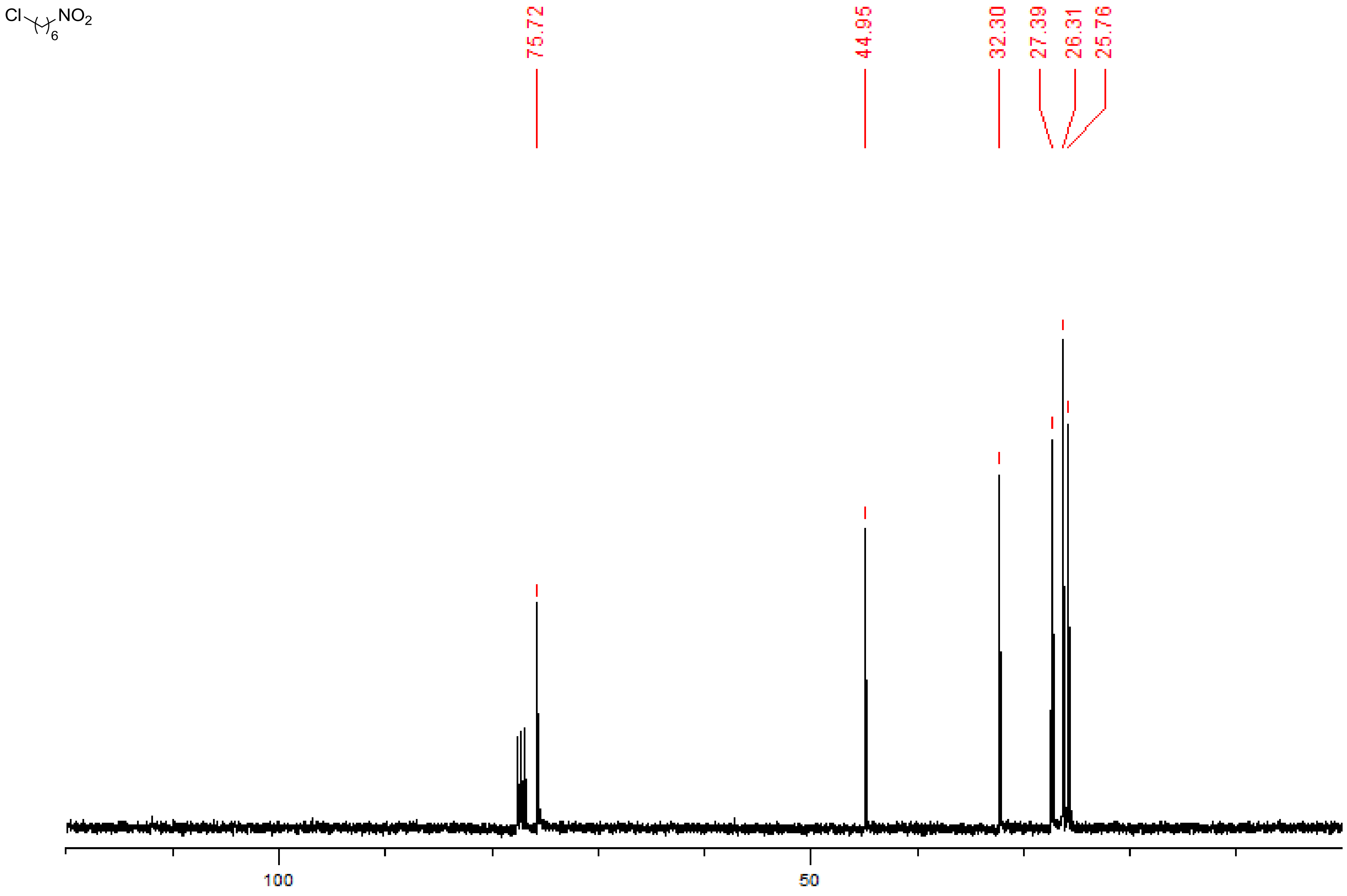

^{1}H NMR - Compound **2h**.

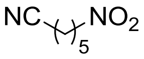

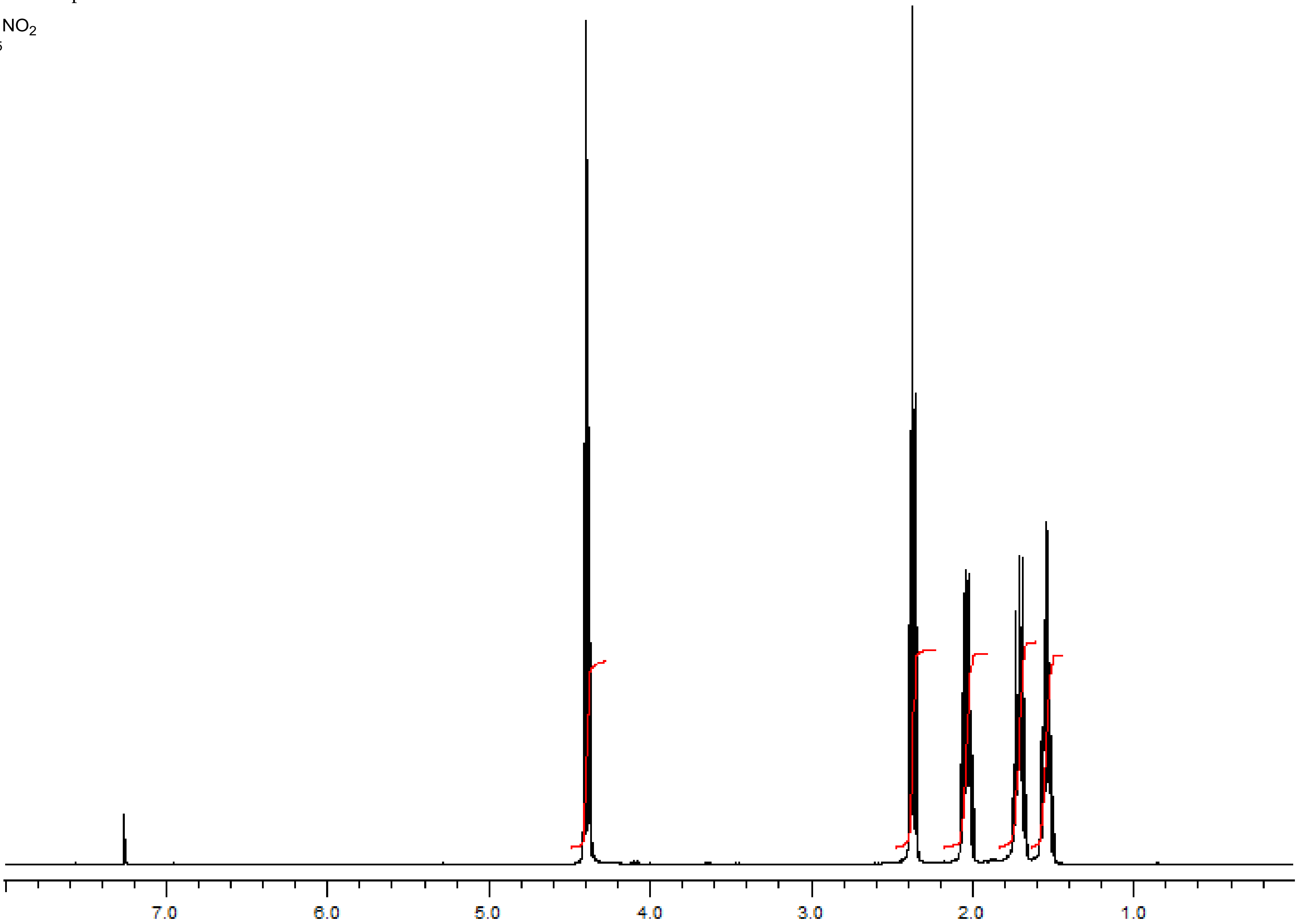

^{13}C NMR - Compound **2h**.

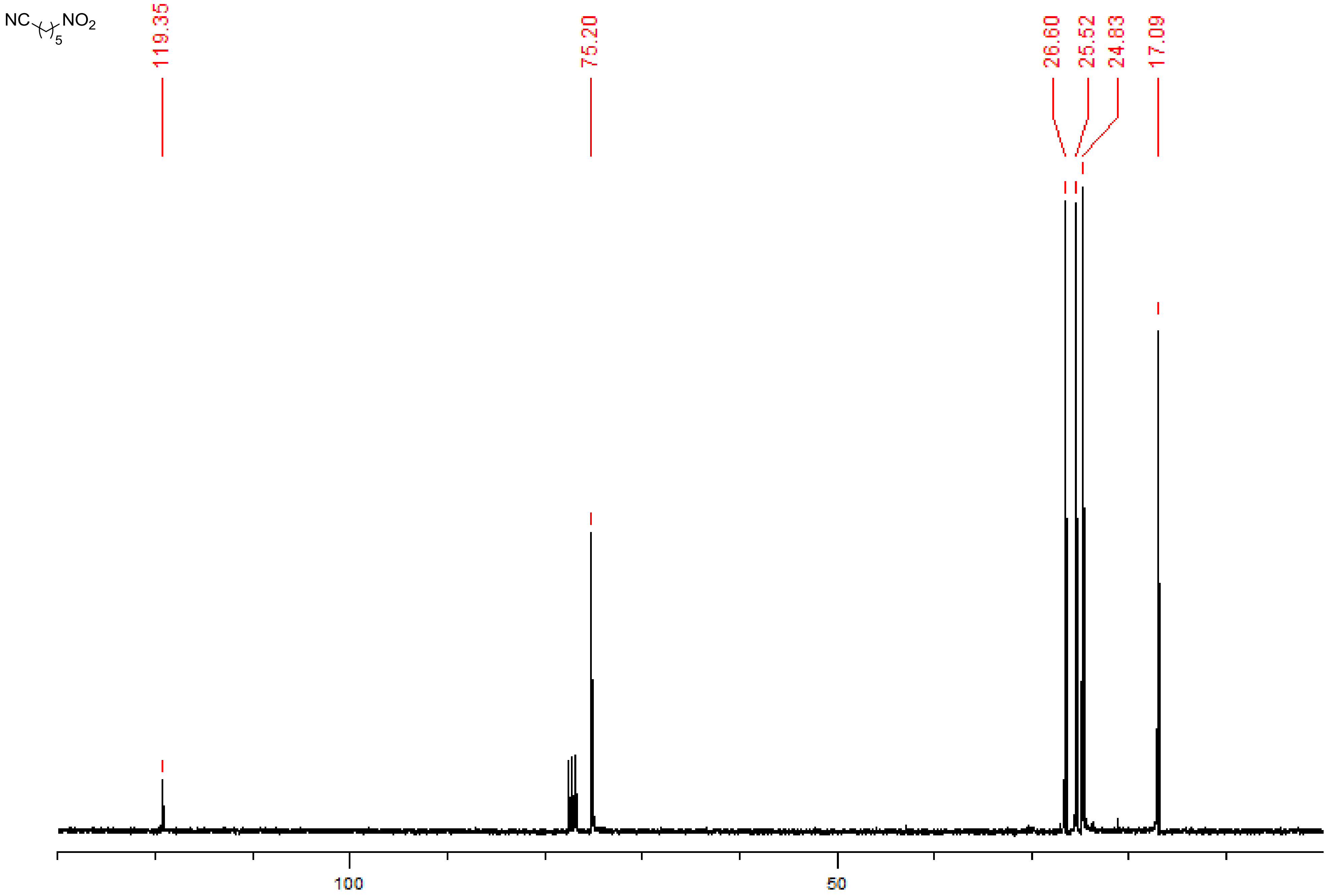

^{1}H NMR - Compound **2i**.

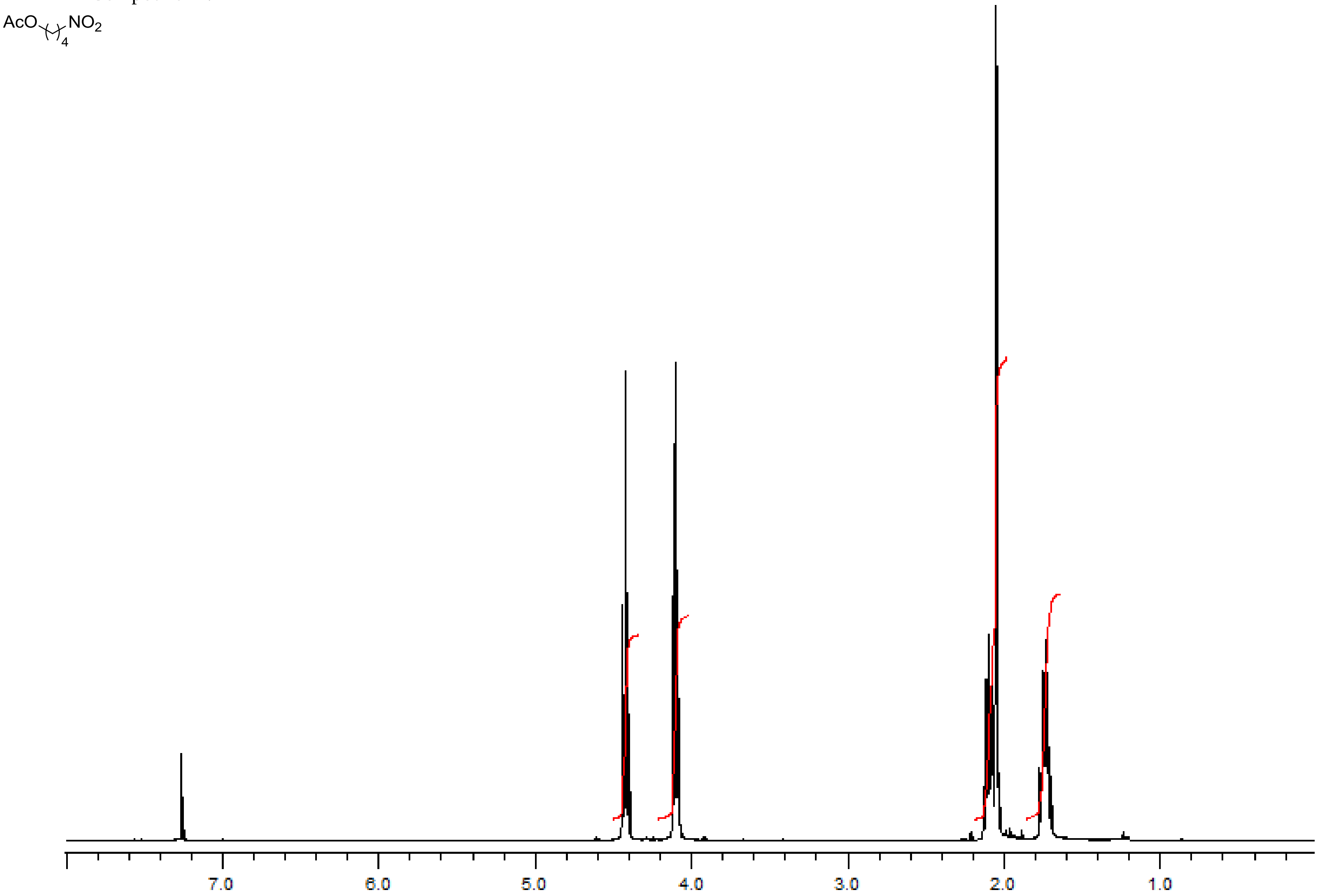

^{13}C NMR - Compound **2i**.

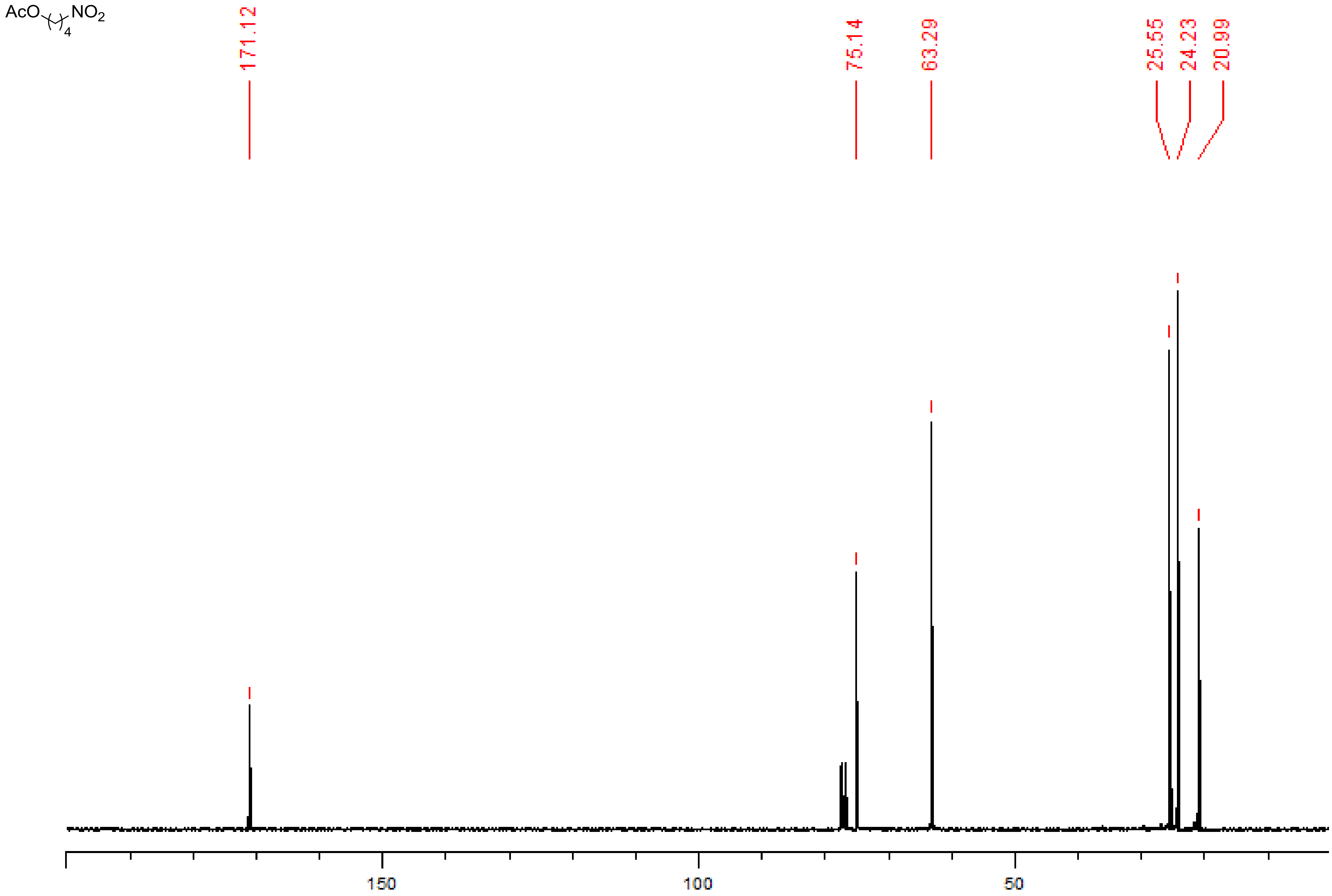

^{1}H NMR - Compound **2j**.

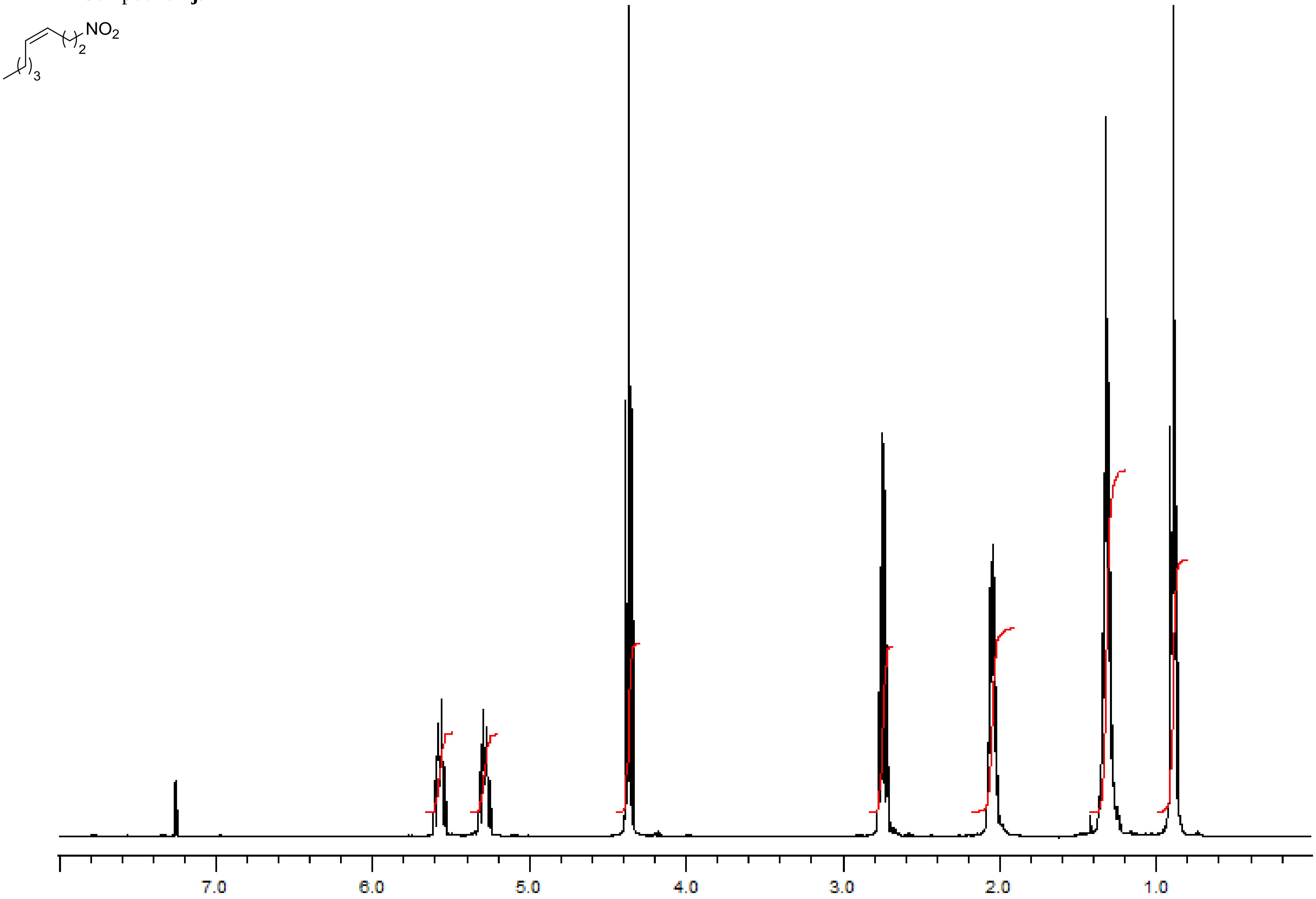

^{13}C NMR - Compound **2j**.

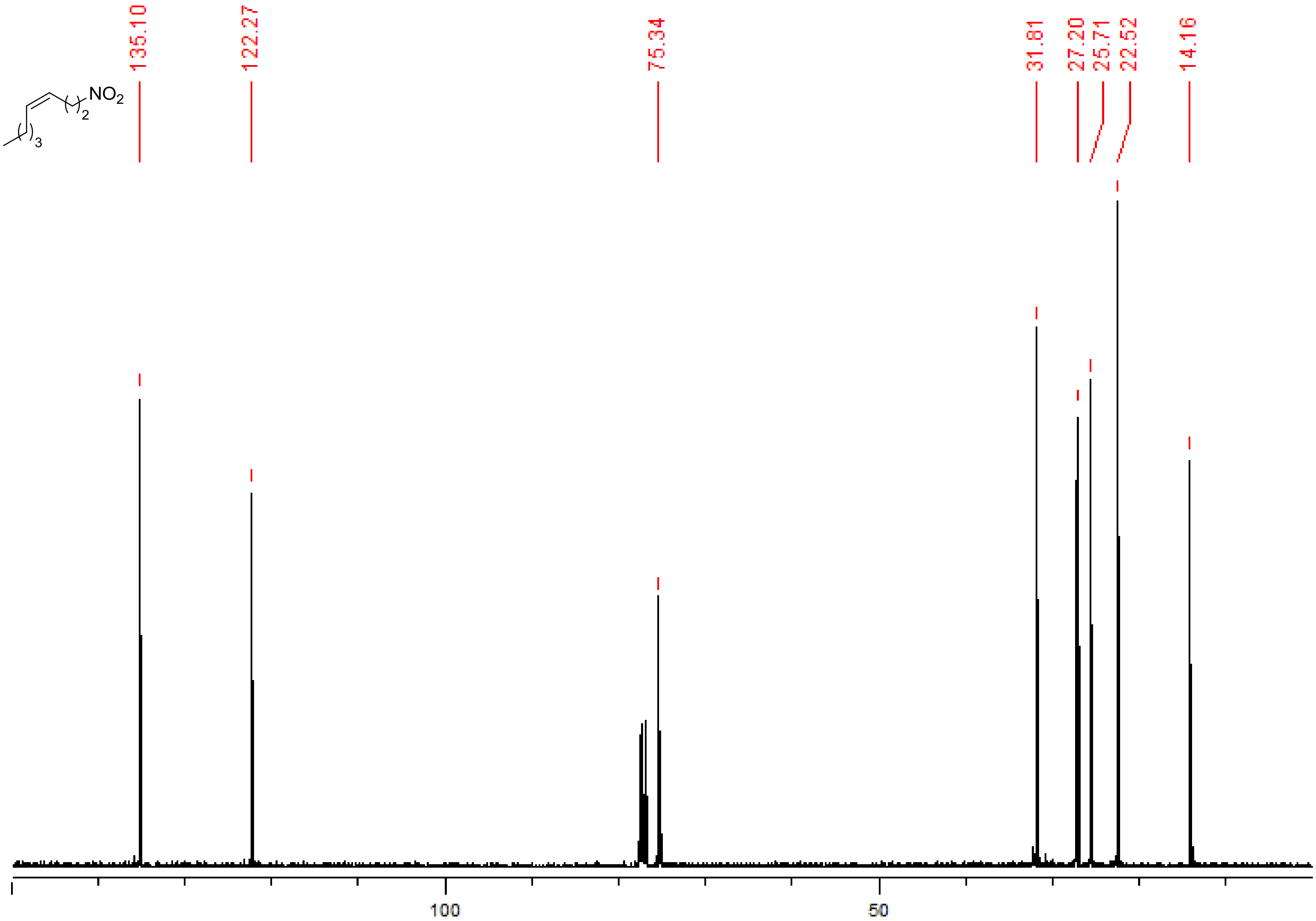

^{1}H NMR - Compound **2k**.

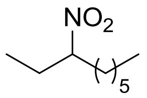

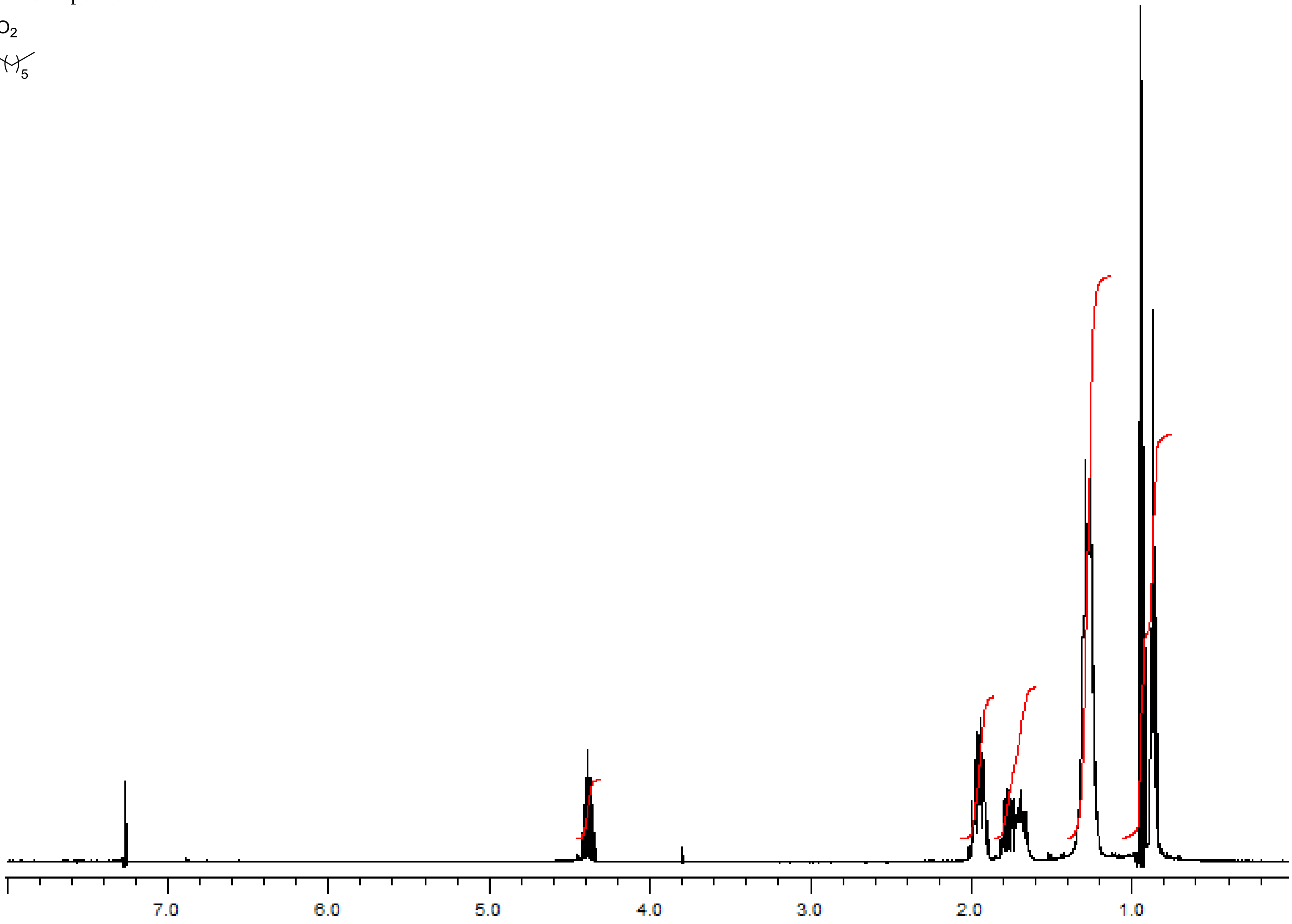

^{13}C NMR - Compound **2k**.

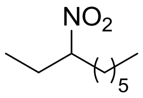

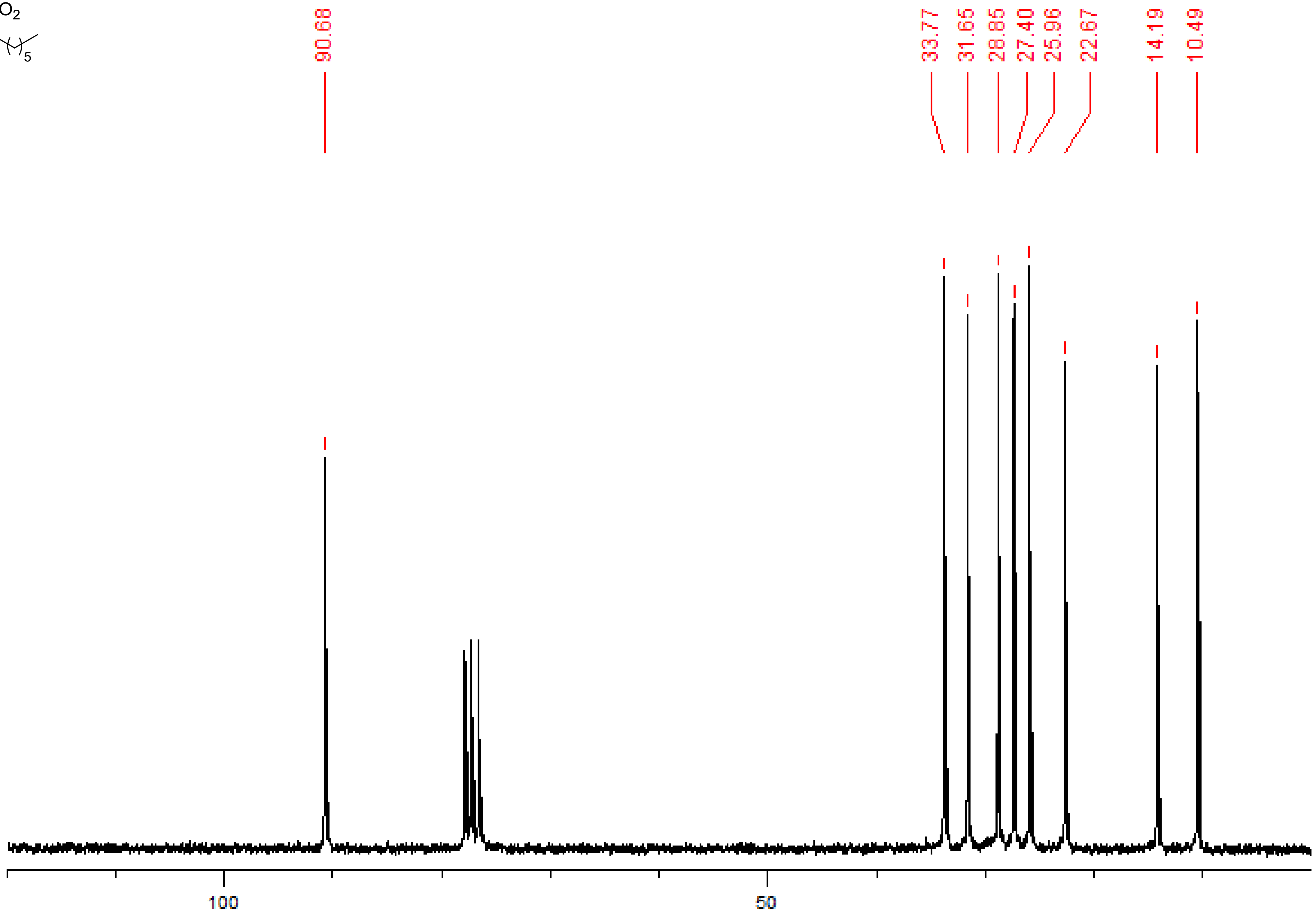

^{1}H NMR - Compound **2l**.

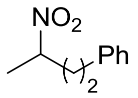

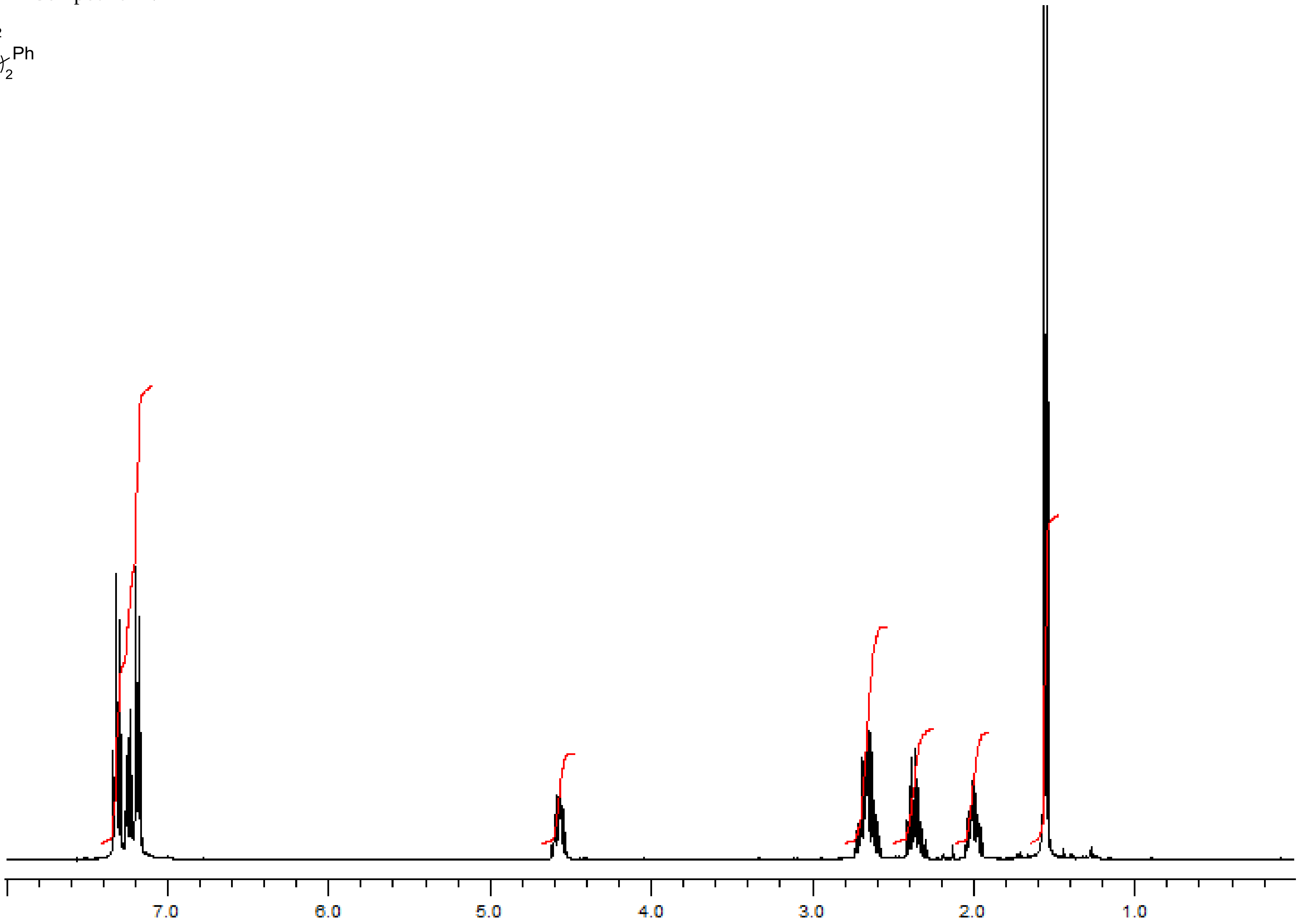

^{13}C NMR - Compound **2l**.

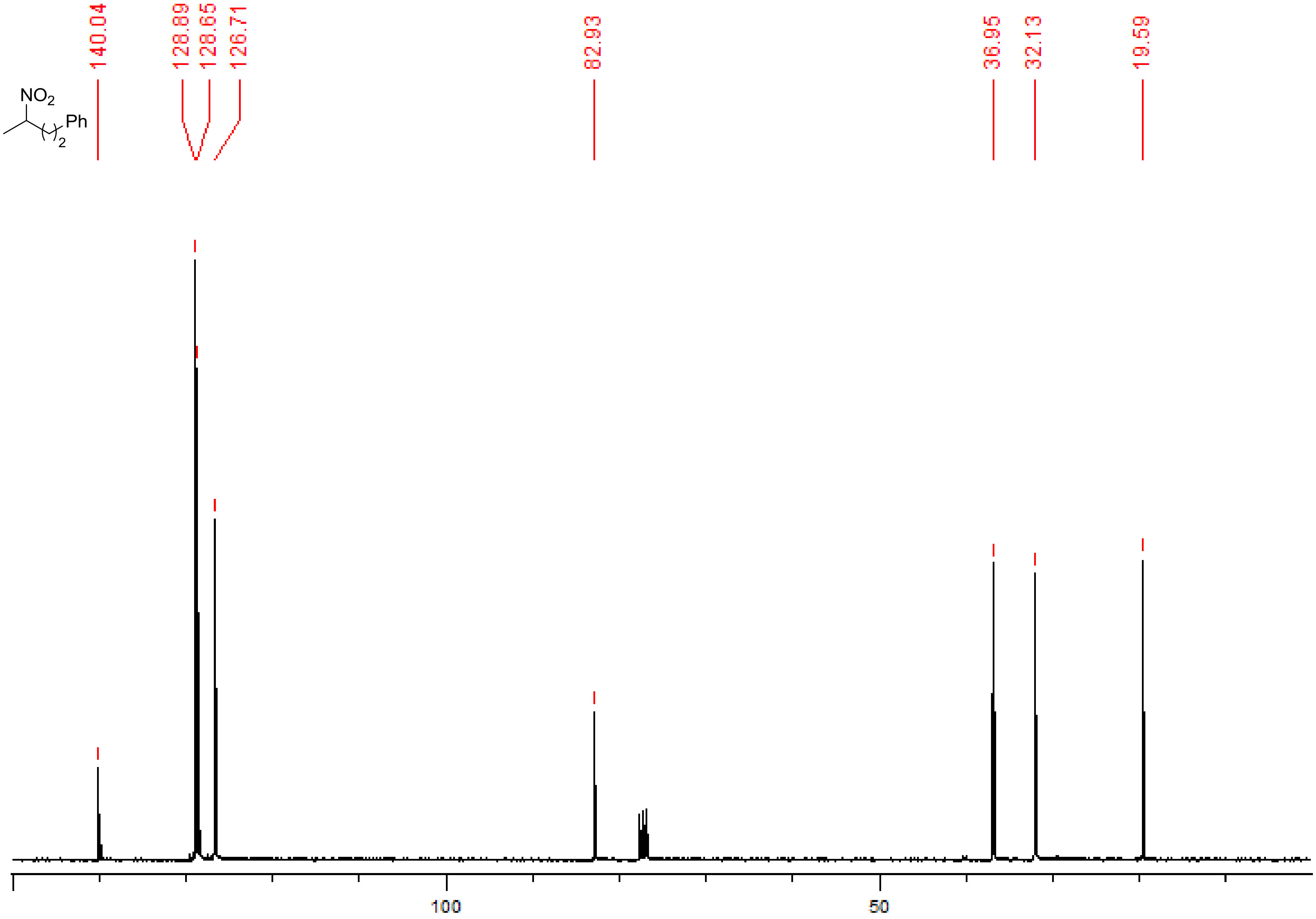

^{1}H NMR - Compound **2m**.

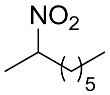

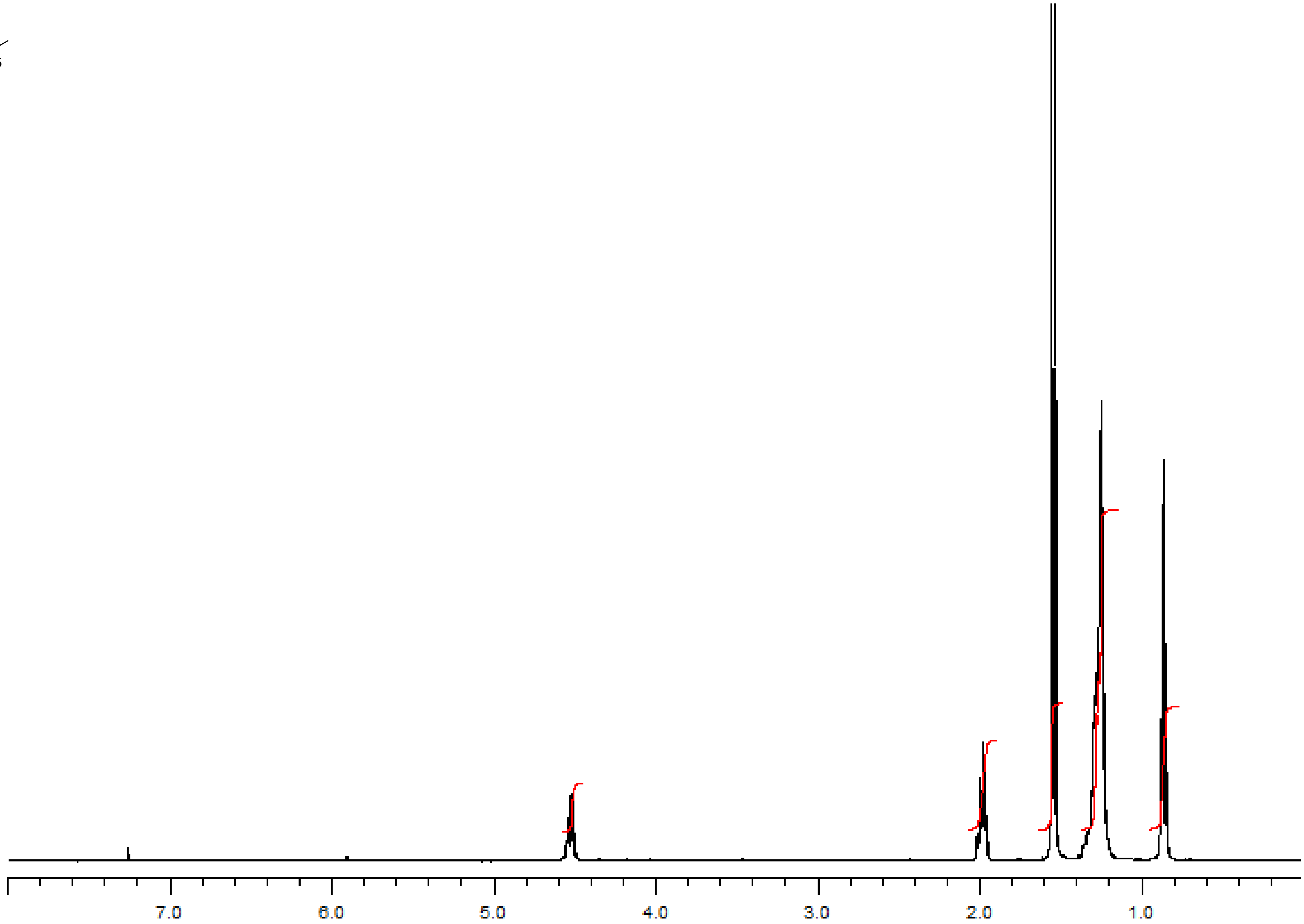

^13^C NMR - Compound **2m**.

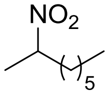

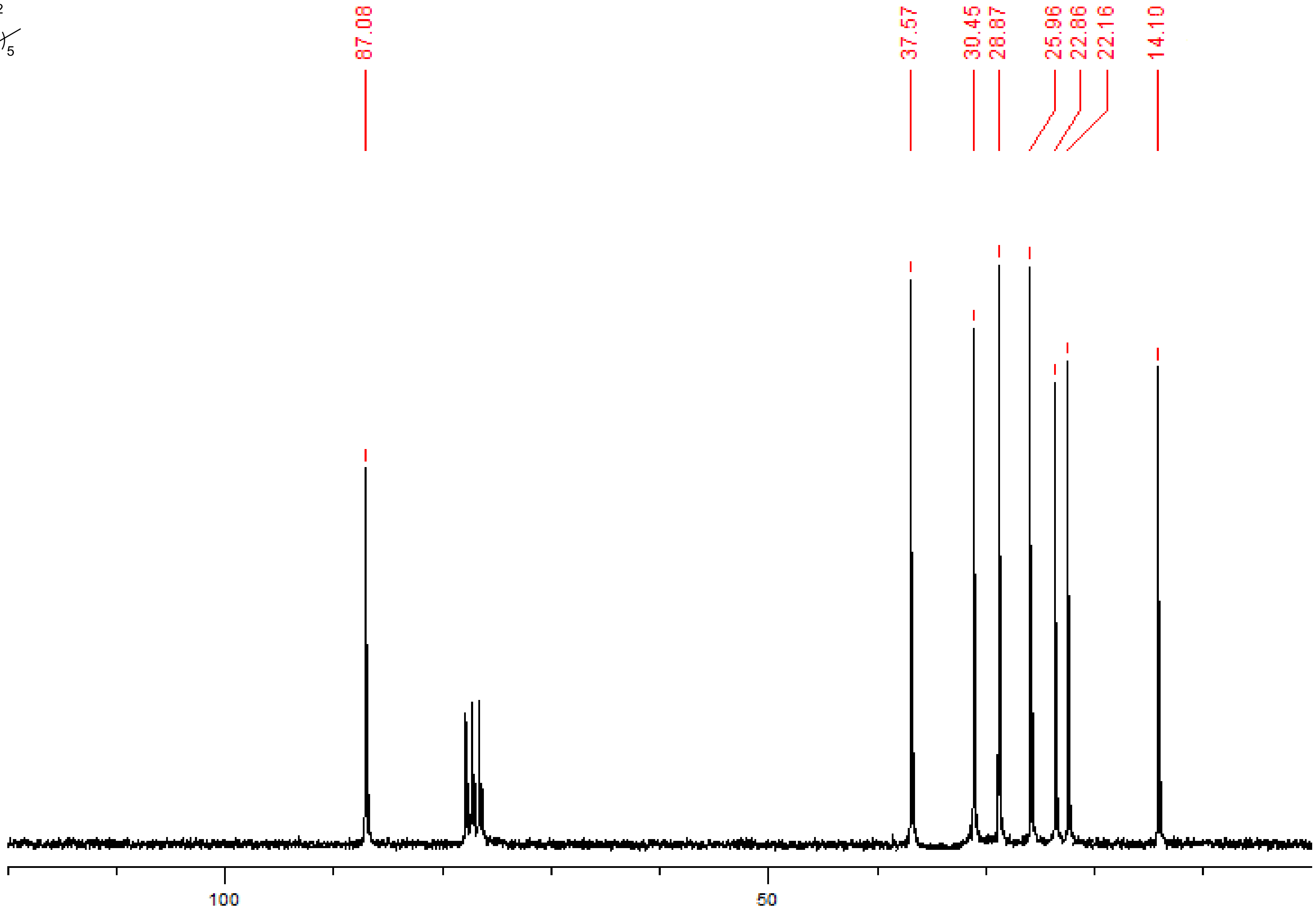

^{1}H NMR - Compound **2n**.

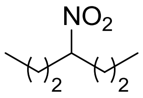

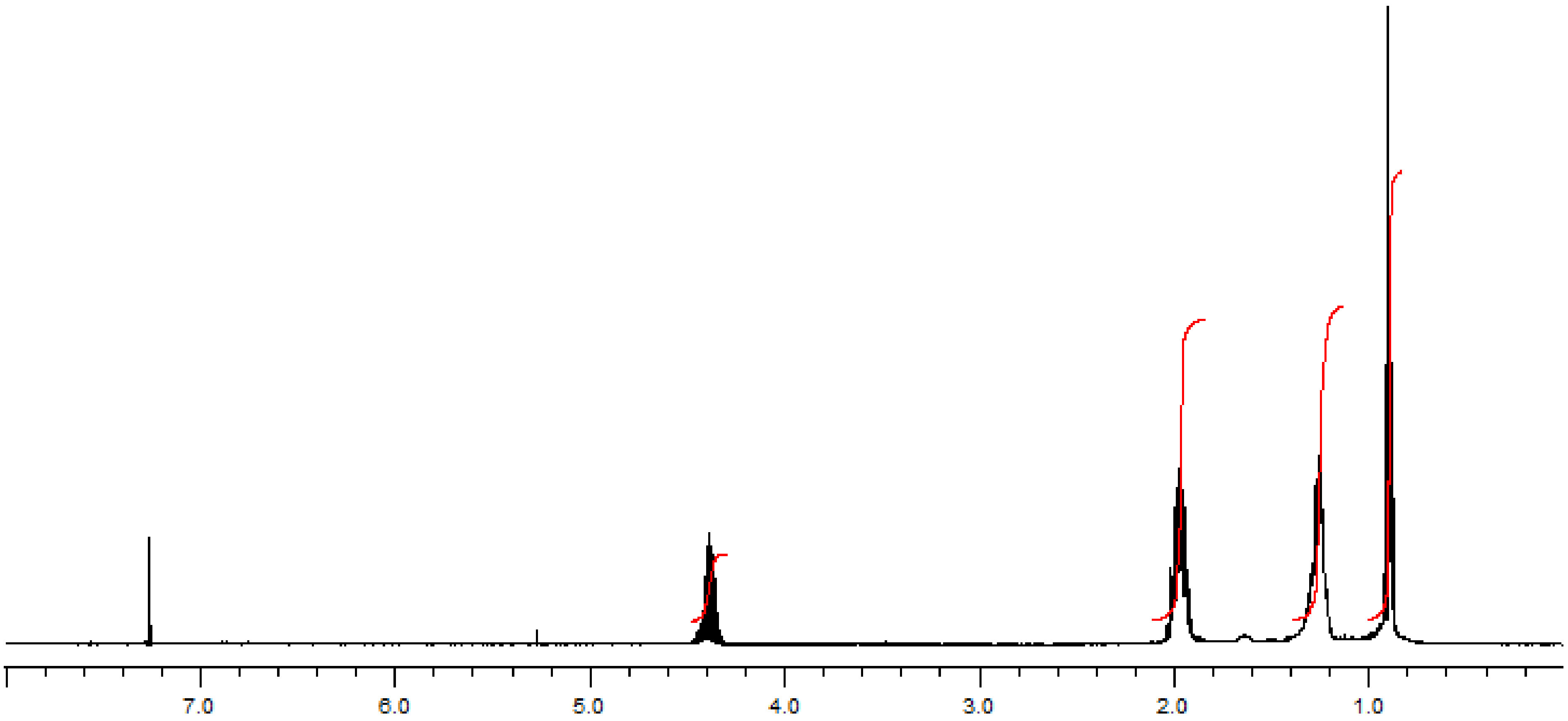

^{13}C NMR - Compound **2n**.

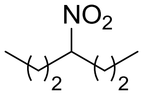

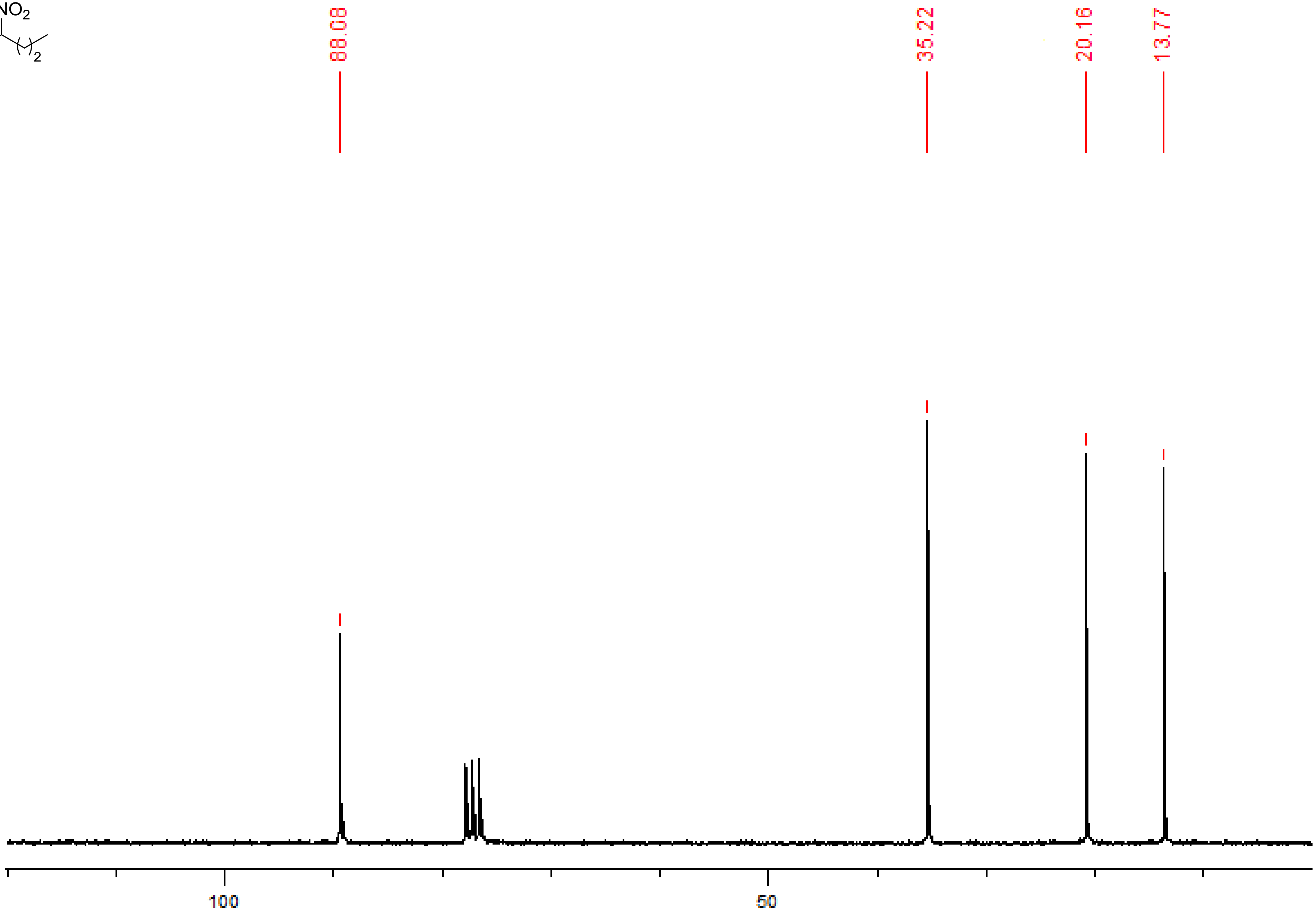

Supplement: File 2 — Copy of 1H and 13C NMR spectra of synthesized compounds. [file Beilstein_J_Org_Chem-09-533-s002.pdf]
